# Constructed Mott–Schottky Heterostructure Catalyst to Trigger Interface Disturbance and Manipulate Redox Kinetics in Li-O_2_ Battery

**DOI:** 10.1007/s40820-024-01476-4

**Published:** 2024-07-29

**Authors:** Yongji Xia, Le Wang, Guiyang Gao, Tianle Mao, Zhenjia Wang, Xuefeng Jin, Zheyu Hong, Jiajia Han, Dong-Liang Peng, Guanghui Yue

**Affiliations:** State Key Lab of Physical Chemistry of Solid Surface, Fujian Key Laboratory of Surface and Interface Engineering for High Performance Materials, College of Materials, Xiamen University, Xiamen, 361005 People’s Republic of China

**Keywords:** Mott–Schottky heterostructure, Lithium-oxygen batteries, Electrocatalysts, Electrodeposition

## Abstract

Lithium-oxygen batteries (LOBs) with high energy density are a promising advanced energy storage technology. However, the slow cathodic redox kinetics during cycling causes the discharge products to fail to decompose in time, resulting in large polarization and battery failure in a short time. Therefore, a self-supporting interconnected nanosheet array network NiCo_2_O_4_/MnO_2_ with a Mott–Schottky heterostructure on titanium paper (TP-NCO/MO) is ingeniously designed as an efficient cathode catalyst material for LOBs. This heterostructure can accelerate electron transfer and influence the charge transfer process during adsorption of intermediate by triggering the interface disturbance at the heterogeneous interface, thus accelerating oxygen reduction and oxygen evolution kinetics and regulating product decomposition, which is expected to solve the above problems. The meticulously designed unique structural advantages enable the TP-NCO/MO cathode catalyst to exhibit an astounding ultra-long cycle life of 800 cycles and an extraordinarily low overpotential of 0.73 V. This study utilizes a simple method to cleverly regulate the morphology of the discharge products by constructing a Mott–Schottky heterostructure, providing important reference for the design of efficient catalysts aimed at optimizing the adsorption of reaction intermediates.

**Supplementary Information:**

The online version contains supplementary material available at 10.1007/s40820-024-01476-4.

## Highlights

- A carbon free self supported Mott-Schottky heterostructure was constructed as an efficient cathode catalyst for lithium oxygen batteries, achieving homogeneous contact between the two materials for strong interfacial interactions.
- The heterostructure triggered interfacial perturbations and band structure changes, which accelerated oxygen reduction reaction (ORR) and oxygen evolution reaction (OER) kinetics, resulting in an extremely long cycle life of 800 cycles and an extremely low overpotential of 0.73 V.
- Combined with advanced characterization techniques and density functional theory calculations, the underlying mechanism behind the boosted ORR/OER activities and the electrocatalytic mechanism were revealed.

**Supplementary Information:**

The online version contains supplementary material available at 10.1007/s40820-024-01476-4.

## Graphical abstract

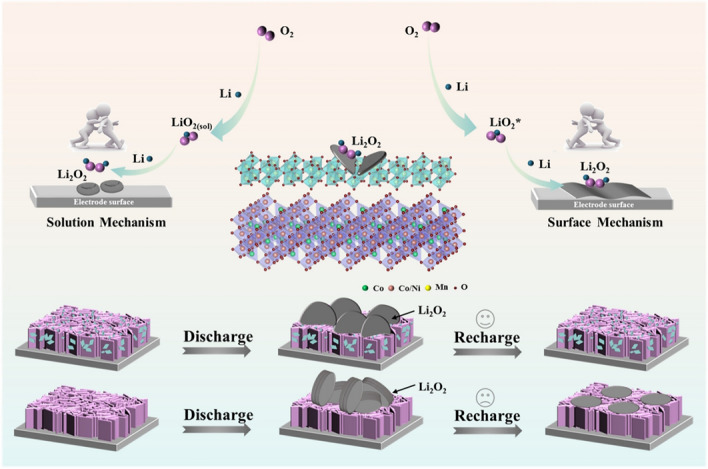

## Introduction

Lithium-oxygen batteries (LOBs) are capable of delivering ultra-high energy density and are expected to fulfill the growing demand for high-energy–density devices in future [1–3]. Non-aqueous LOBs [4, 5] face bottlenecks such as poor rate performance, low practical capacity and short cycle life [6–8] due to the slow three-phase (gas/solid/liquid) reaction, the stacking of the insulated discharge product on the cathode side, as well as parasitic reactions caused by attacks from superoxide radicals and singlet oxygen (^1^O_2_) in the electrolyte [1, 4, 9–11]. Among them, the adsorption and morphology of the discharge products/intermediates at the cathode side are important factors affecting the above issues [12]. The adsorption capacity of the reaction intermediates is inextricably linked to the overpotential, the cycling reversibility, and the capacity [13–15]. Therefore, finding effective strategies to regulate the adsorption of discharge products/intermediates is the ultimate goal of designing efficient electrocatalysts.

Designing multiphase interfaces and regulating the active site distribution of electrocatalysts may be an effective strategy to induce transformative changes in oxygen reduction (ORR) and oxygen evolution (OER) activity and intermediate sorption energy. When two materials with different work functions contact each other, electron redistribution will occur at the heterogeneous interface to adjust the Fermi level of adjacent components, known as the Mott–Schottky effect [16], to establish a robust built-in electric field [17] to promote electron transfer. The heterogeneous interface will alter the work function and electron cloud density of the material, enhancing the adsorption of oxygen and desorption the products. The Mott–Schottky heterostructures have been used as a catalyst for lithium-sulfur batteries and Li-CO_2_ batteries and have demonstrated excellent catalytic activity [16, 18–23]. In addition, the interface disturbance caused by the heterostructure can effectively change the charge distribution of the active site [24, 25], thus regulating the morphology of Li_2_O_2_ [26]. For example, Yin et al. [27] designed a NiCo_2_S_4_/NiO heterostructure cathode catalyst in which the discharge product Li_2_O_2_ grows into a large size pea-like shape, endowing the electrode with an extremely high discharge capacity of up to 10,050 mAh g^−1^ and a tiny overpotential of 0.88 V. Similarly, Yan et al. designed a hierarchical heterostructure MnO_2_-Co_3_O_4_ to achieve embedded growth of bulk Li_2_O_2_ [28]. The embedded large mooncake and flake Li_2_O_2_ provide enhanced cycle stability, excellent rate performance and high capacity. Therefore, it is feasible to carefully select appropriate materials to construct reasonable heterogeneous structures to regulate work functions and achieve high performance LOBs. However, achieving uniform contact between the two materials to obtain strong interfacial interactions is a challenge, which to some extent limits the capacity and battery life of LOBs. Furthermore, there is a lack of studies on the heterogeneous structure and intermediate adsorption behavior, as well as the distribution and formation mechanism of discharge products.

In this work, a facile electrodeposition combined with hydrothermal method was employed to achieve the growth of uniform vertically interconnected arrays of NiCo_2_O_4_ nanosheets attached with small flakes of MnO_2_ on the surface of smooth titanium paper (TP-NCO/MO). The design of self-supporting structures suppresses the aggregation of catalyst materials, enabling uniform contact between the two materials and exposing more catalytic active sites. Electron energy loss spectroscopy (EELS) and other characterization confirm that the construction of NiCo_2_O_4_/MnO_2_ heterostructure triggers the interface disturbance at the heterogeneous interface and induces the interface charge reconstruction, thus adjusting the adsorption energy of the intermediate and promoting the product decomposition. Density functional theory (DFT) calculations demonstrate that LiO_2_ adsorbs weakly on NiCo_2_O_4_/MnO_2_. The weak adsorption of the intermediate product LiO_2_ within an optimal range facilitates the swift conversion of the intermediate to the final product, accelerating the kinetics of discharge product formation. Ultimately, the LOB assembled with TP-NCO/MO cathode exhibited over 800 cycles of cycling performance, low overpotential of 0.73 V and excellent rate capability. A series of characterizations revealed that the discharge products in the TP-NCO/MO electrode had high reversibility. These results serve as a valuable guide for creating efficient LOBs catalysts.

## Experimental Section

### Preparation of TP-NCO/MO

A facile electrodeposition was adopted to prepare NiCo_2_O_4_ nanosheets on TP. Before deposition, TP was first soaked in HNO_3_ for 8 h to clean the surface and remove the oxide layer. The solution of 0.3 M Co(NO_3_)_2_⋅6H_2_O and 0.15 M Ni(NO_3_)_2_⋅6H_2_O was synthesized as the electrolyte, while Pt sheet and 4 × 4 cm^2^ TP were utilized as counter/reference electrode and working electrode, respectively. An electrochemical station was utilized to apply a constant current of 10 mA cm^−2^ for 15 min. After deposition, samples were washed with DI water and ethanol and then held in muffle furnace at 300 °C for 3 h to obtain the TP-NCO. The as-prepared TP-NCO was placed in 70 mL aqueous solution containing 0.1106 g KMnO_4_ and 40 µL H_2_SO_4_ and maintained at 80 °C for 1 h to perform hydrothermal reaction. After washing, the sample was calcined at 400 °C in the air for 4 h. The catalyst TP-NCO/MO was obtained after cooling to room temperature. The chemical reaction equations involved are as follows:

1 $${\text{H}}_{2} {\text{O}} + 2e^{ - } + {\text{NO}}_{3}^{ - } \to {\text{NO}}_{2 }^{ - } + 2{\text{OH}}^{ - }$$

2 $$6{\text{H}}_{2} {\text{O}} + 6e^{ - } + {\text{NO}}_{2}^{ - } \to {\text{NH}}_{4 }^{ + } + 8{\text{OH}}^{ - }$$

3 $$x{\text{Ni}}^{2 + } + 2x{\text{Co}}^{2 + } + 6x{\text{OH}}^{ - } \to {\text{Ni}}_{x} {\text{Co}}_{2x} ({\text{OH}})_{6x}$$

4 $${\text{Ni}}_{x} {\text{CO}}_{2x} ({\text{OH}})_{6x} + 0.5x{\text{O}}_{2} \to x{\text{NiCo}}_{2} {\text{O}}_{4 } + 3x{\text{H}}_{2} {\text{O}}$$

5 $$4{\text{H}}^{ + } + 3e^{ - } + {\text{MnO}}_{4}^{ - } \to {\text{MnO}}_{2} + 2{\text{H}}_{2} {\text{O}}$$

### Material Characterization

The phase structure of catalyst materials was determined by X-ray diffraction (XRD, Bruker AXS) with Cu-kα radiation at 40 kV and 40 mA. The surface morphology and microstructure of the materials were observed using scanning electron microscopy (SEM, SU70, Hitachi) and transmission electron microscopy (TEM, TECNAI F-30). Electron energy loss spectroscopy (EELS) was performed by JEOL JEM-F200 (operating voltage 200 kV). Raman spectra were recorded by a HR Evolution high-resolution confocal laser Raman system (France, Horiba FRANCE SAS) with a wavelength of 532 nm. N_2_ adsorption/desorption analysis curves were obtained by a 3H-2000PM2 analyzer. The specific surface area and pore size distribution curves of the material were obtained using the BJH model. The X-ray photoelectron spectroscopy (XPS) were recorded on Thermo Scientific K-Alpha spectrometer (USA). FTIR spectroscopy was measured on a Fourier infrared spectrometer (Nicolet IS50). UV–vis diffuse reflectance spectroscopy was obtained with Shimadzu UV-3600i Plus of Japan. TOF–SIMS was performed on an IONTOF M6, and in situ EIS was carried out on a PARSTAT 3000A-DX electrochemical workstation (AMETEK Instrument Co., Ltd.).

### Electrochemical Measurements

The prepared TP-NCO/MO and TP-NCO were cut into electrodes with a diameter of 12 mm, which were directly used as the cathode of LOBs. Lithium foil with a thickness of 0.6 mm was used as a counter electrode, and glass fiber (Whatman, GF/D) was used as the diaphragm, the electrolyte was 1 M LiTFSI in TEGDME (Canrd Technology Co., Ltd.). The 2032 battery was assembled in the Ar atmosphere glove box and was transferred to the glass test bottle filled with O_2_ for battery testing after a period of standing. The constant current charge and discharge tests were conducted on a NEWARE BTS multi-channel battery system (Shenzhen, China) with a voltage range set at 2.0 − 4.8 V (vs Li^+^/Li). The MnO_2_ and Super P oxygen cathodes were obtained by mixing and stirring the catalyst material (MnO_2_ /Super P): CNT: polyvinylidene fluoride (PVDF) at a ratio of 4:4:2 and coated on a 12 mm carbon paper. The rest is the same as the above process. Cyclic voltammetry (CV) and electrochemical impedance spectroscopy (EIS) were recorded by CHI660D electrochemical workstation. CV was performed at a sweep rate of 0.1 mV s^−1^ in the voltage range of 2.0–4.5 V (vs Li^+^/Li), and EIS testing was performed in the frequency range of 10^5^ to 0.01 Hz and a sine wave of 5 mV. The band edge potential of the catalysts was measured by the Mott–Schottky test using a Pt counter electrode, an Ag/AgCl reference electrode and a catalyst working electrode in 0.5 M Na_2_SO_4_ solution at a frequency of 2000 Hz.

### DFT Calculations

Vienna Ab initio Simulation Package (VASP) was used to perform the first-principles calculation, using the projector-augmented wave method based on DFT [29]. The generalized gradient approximation (GGA) of the Perdew–Burke–Ernzerhof (PBE) exchange–correlation functional was selected for calculation [30]. The correction of van der Waals interaction adopted the Grimme DFT-D3 method [31]. A plane-wave (PW) cutoff energy of 500 eV was set. The relaxation and self-consistent calculation were based on the 3 × 3 × 1 k-point mesh generated by the Monkhorst–Pack (M–K) scheme. The convergence criteria are 1 × 10^−5^ eV energy differences for solving the electronic wave function, and the structures were relaxed until the forces on all atoms were smaller than − 0.02 eV Å^−1^, while the perovskite lattice parameters and the bottom two-layer atoms were fixed. A vacuum layer over 15 Å along the z-direction was provided to eliminate direct interaction between adjacent plates. A post-stage van der Waals DFT-D3 method with Becke-Johnson damping was applied [31], and the Hubbard-U corrections to the d-electrons of Ru and Ir following the approach proposed by Dudarev et al. [32] were considered. The values of the effective Hubbard-U parameter U = 3.3, 3.9, and 6.2 eV are chosen for Co, Mn, and Ni, respectively, determined by linear response approach [33].

In practice, NiCo_2_O_4_ on MnO_2_ may exist in a variety of crystalline surface combinations. We found that the low-index facet of NiCo_2_O_4_ has a good matching with MnO_2_, and the interfacial strain is lower than 1.5%, from which MnO_2_ can be easily epitaxial grown on this crystalline facet. We adopt this model as the mechanism study in the calculation.

The possible reaction paths of Li^+^ and O_2_ on the surface of NiCo_2_O_4_/MnO_2_:

4e^−^ pathway:

6 $${\text{Li}}^{ + } + e^{ - } + {\text{O}}_{2} \to {\text{LiO}}_{2}^{*}$$

7 $${\text{LiO}}_{2}^{* } + {\text{Li}}^{ + } + e^{ - } \to {\text{Li}}_{2} {\text{O}}_{2}^{*}$$

8 $${\text{Li}}_{2} {\text{O}}_{2}^{*} + {\text{Li}}^{ + } + e^{ - } \to {\text{Li}}_{3} {\text{O}}_{2}^{*}$$

9 $${\text{Li}}_{3} {\text{O}}_{2}^{*} + {\text{Li}}^{ + } + e^{ - } \to {\text{Li}}_{4} {\text{O}}_{2}^{*}$$

2e^−^ pathway:

10 $${\text{Li}}^{ + } + e^{ - } + {\text{O}}_{2} \to {\text{LiO}}_{2}^{*}$$

11 $${\text{LiO}}_{2}^{* } + {\text{Li}}^{ + } + {\text{e}}^{ - } \to {\text{Li}}_{2} {\text{O}}_{2}^{*}$$

12 $${\text{Li}}_{2} {\text{O}}_{2}^{*} + {\text{Li}}^{ + } + e^{ - } + {\text{O}}_{2} \to {\text{Li}}_{3} {\text{O}}_{4}^{*}$$

13 $${\text{Li}}_{3} {\text{O}}_{4}^{*} + {\text{Li}}^{ + } + {\text{e}}^{ - } \to {\text{Li}}_{4} {\text{O}}_{4}^{*}$$

## Results and Discussion

### Synthesis and Characterization of Catalysts

**Fig. 1:**
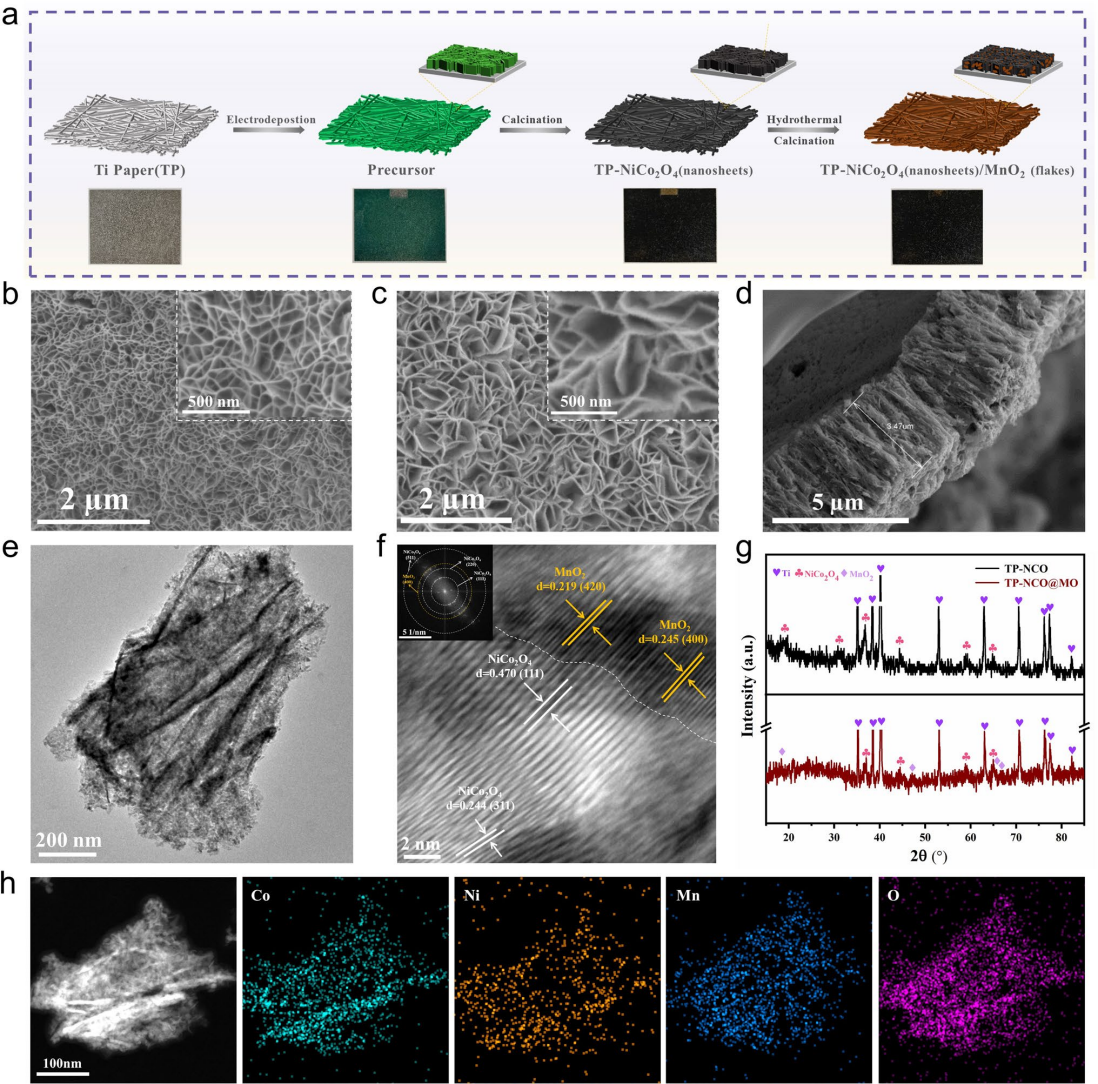
**a** Flow illustration of TP-NCO/MO preparation. SEM pattern of **b** TP-NCO and **c** TP-NCO/MO array (inset shows its further enlargement). **d** Cross-sectional SEM image of TP-NCO/MO.** e** TEM pattern of NCO/MO array. **f** HRTEM pattern of NCO/MO. (inset shows the corresponding FFT pattern) **g** XRD patterns of TP-NCO/MO and TP-NCO. **h** Elemental mapping of NCO/MO

Figure 1a outlines the preparation process of TP-NCO/MO. Initially, the precursor nanosheet arrays were vertically and uniformly deposited on the surface of titanium fiber using a facile electrodeposition technique. During this process, oxynitrate anions on the cathode side were reduced to form hydroxide. The synthesized metal hydroxide precursor was calcined in air to obtain NiCo_2_O_4_. Subsequently, small MnO_2_ flakes were grown on the surface of NiCo_2_O_4_ nanosheets through hydrothermal and calcination processes. SEM images were obtained to reveal the surface morphology of the materials. The comparative views of the TP-NCO and TP-NCO/MO nanosheet arrays are clearly shown in Fig. 1b, c. Notably, small and thin flakes of MnO_2_ nanosheets grow on the surface of TP-NCO/MO nanosheets, while the surface of TP-NCO nanosheets appears smooth. The presence of MnO_2_ flakes does not block the original pore channels, as evidenced by the cross-sectional SEM image of TP-NCO/MO in Fig. 1d. Figure 1d also shows that the nanosheet arrays are arranged vertically aligned on the substrate and are tightly connected to the substrate without obvious gaps. Rough measurements indicate that the thickness of the TP-NCO/MO nanosheet array is approximately 3.47 µm. The elemental mapping images in Fig. S7 illustrate that the Co, Ni, Mn, and O elements are uniformly distributed across the titanium fiber. The presence of MnO_2_ can be demonstrated by TEM. The morphology of NCO/MO nanosheet arrays scraped from TP is shown in Fig. 1e. It can be seen that the nanosheet arrays are transparent, indicating their ultra-thin characteristics and the existence of inter-nanosheet channels, which aligns with the conclusions drawn from SEM. The high-resolution TEM (HRTEM) image demonstrates a distinct heterogeneous interface in the material (Fig. 1f). The lattice stripes, with a lattice spacing of 0.470 and 0.244 nm below the interface, correlate to the (111) and (311) crystal planes of NiCo_2_O_4_, respectively. The lattice fringes above the interface correspond to the (420) and (400) crystal planes of MnO_2_, respectively. The HRTEM conclusion is supported by the corresponding fast Fourier transform (FFT) results in the illustration. The results further confirm that MnO_2_ was successfully grown on the surface of NiCo_2_O_4_ nanosheets, forming a close contact to create a heterogeneous interface. The elemental distribution on the side of the nanosheets array was revealed by the high-angle annular dark-field scanning TEM (HAADF-STEM) and corresponding elemental mappings in Fig. 1h. The image showed that Co and Ni elements were more concentrated in the branches of the nanosheet. These findings were consistent with the SEM results, indicating the growth of MnO_2_ on the surface of NiCo_2_O_4_ nanosheets. The phases of TP-NCO/MO and TP-NCO were further characterized by XRD tests (Fig. 1g). The pronounced diffraction peaks in TP-NCO can be indexed to the substrate Ti (JCPDS No. 65–3362) and spinel-type NiCo_2_O_4_, respectively. The intense diffraction peaks of Ti cover the peaks of other phases to a certain extent, so they were treated as breakpoints. Specifically, the diffraction peaks situated at 18.9°, 31.1°, 36.7°, 44.6°, 59.1°, and 65.0° can be retrieved as the (111), (220), (311), (400), (511), and (440) crystal planes of NiCo_2_O_4_ (JCPDS No. 20–0781), respectively. For TP-NCO/MO, except for the diffraction peaks associated with Ti and NiCo_2_O_4_, the diffraction peaks at 36.7°, 37.5°, 47.4°, 60.3°, and 65.1° correspond to the (400), (211), (510), (521), and (002) crystal planes of α-MnO_2_ (JCPDS No. 44–0141), respectively, where the diffraction peaks at 36.7° and 65.1° basically coincide with NiCo_2_O_4_. The Raman results can further confirm the presence of NiCo_2_O_4_ and MnO_2_ in the catalyst material (Fig. S10). According to literature reports, MnO_2_ has a variety of crystal structures, such as α-MnO_2_, β-MnO_2_ γ-MnO_2_, δ-MnO_2_, and ε-MnO_2_ [34–38]. Among them, the essential structural unit of α-MnO_2_ consists of [MnO_6_] octahedral double chains, which are intertwined in the plane with adjacent double chains to form a (2 × 2) + (1 × 1) tunnel structure [39]. The tunnel structure can promote the transport of discharge products, Li^+^ and O_2_, improve the formation and breakdown efficiency of discharge products, and degrade overpotential.

### Exploration of Heterogeneous Interfaces

**Fig. 2:**
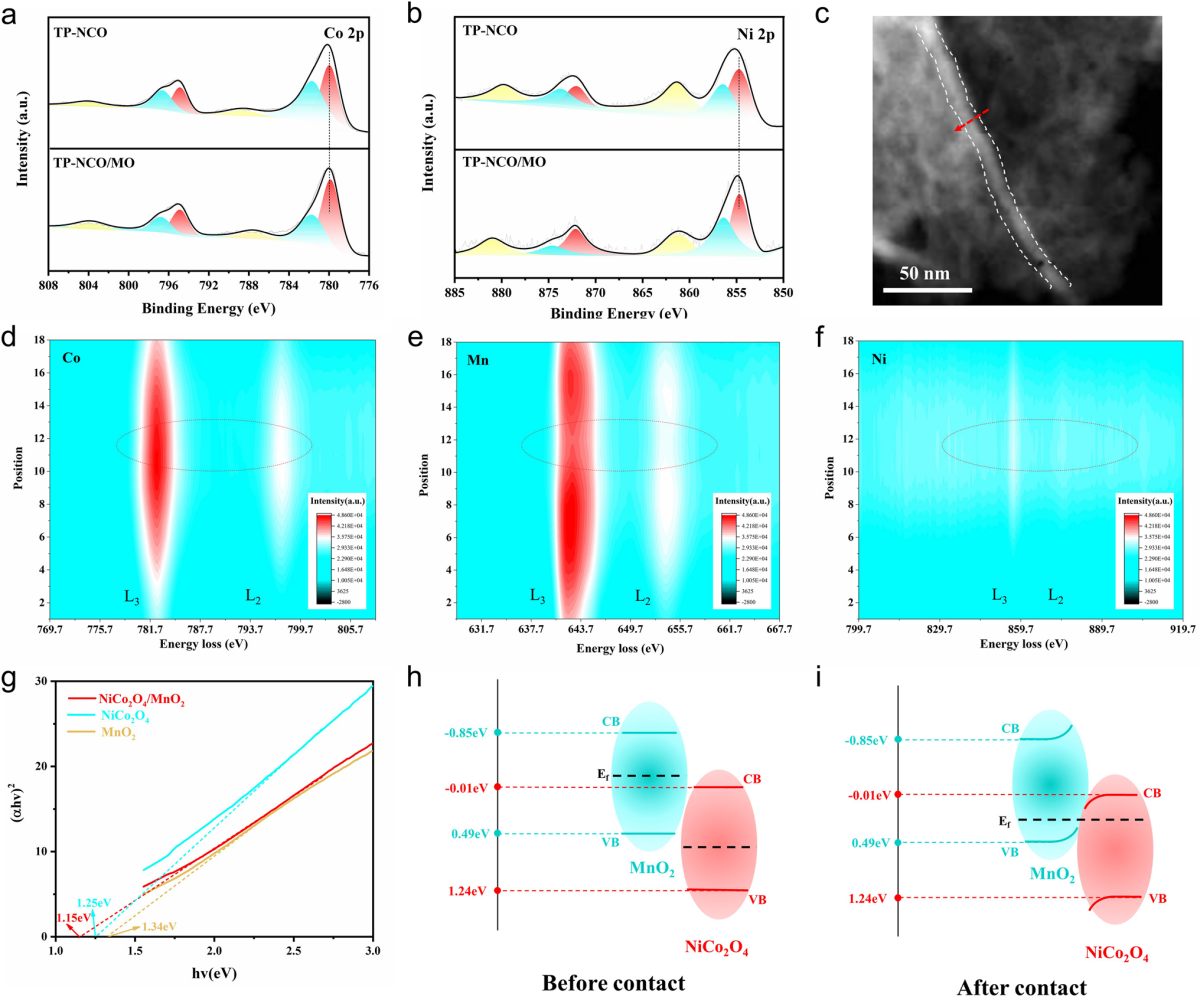
High-resolution XPS spectrum of **a** Co 2*p* and **b** Ni 2*p* of TP-NCO/MO and TP-NCO. **c** STEM image and EELS scanning direction (red arrow). EELS spectra of **d** Co L_2,3_-edges, **e** Mn L_2,3_-edges and **f** Ni L_2,3_-edges. **g** Bandgap of the NiCo_2_O_4_/MnO_2_, NiCo_2_O_4_ and MnO_2_ calculated from the plots of (αhν)^2^. versus the photon energy (hν). Possible energy band diagrams of **h** before contact and **i** after contact for NiCo_2_O_4_ and MnO_2_

XPS was utilized to acquire the information on possible electronic structure alterations, elemental composition and chemical environment of the catalyst materials. The full spectrum of TP-NCO/MO shows the presence of Co, Ni, O, and Mn signals (Fig. S11a), with the elemental contents of 5.96%, 2.92%, 64.47%, and 26.65%, respectively (Fig. S12), and the ratio of Co and Ni elementals is close to 2:1, which is consistent with the expected results. Figure 2a shows the high-resolution fine spectra of TP-NCO@MO and TP-NCO on Co 2*p*. For TP-NCO@MO, the characteristic peaks at 779.75 and 794.86 eV link to Co^3+^ 2*p*_3/2_ and Co^3+^ 2*p*_1/2_, whereas the peaks at 781.65 and 796.75 eV are associated with Co^2+^ 2*p*_3/2_ and Co^2+^ 2*p*_1/2_, 787.53 and 803.82 eV are considered satellite peaks. In contrast to TP-NCO, TP-NCO@MO has a slight displacement toward low binding energy. Regarding the high-resolution spectrum for Ni 2*p* (Fig. 2b), in TP-NCO@MO, the peaks at 854.69 and 872.13 eV belong to Ni^2+^ 2*p*_3/2_ and Ni^2+^ 2*p*_1/2_, 856.37 and 874.58 eV correspond to Ni^3+^ 2*p*_3/2_ and Ni^3+^ 2*p*_1/2_, 861.25 and 881.01 eV are considered satellite peaks. Compared with TP-NCO, Co 2*p* and Ni 2*p* are negatively shifted in TP-NCO@MO, which suggests that the presence of MnO_2_ induces a change in the bonding environment [40], indicating a strong electronic interaction between NiCo_2_O_4_ and MnO_2_, which is an electronic coupling effect induced by the establishment of a heterogeneous interface. Reasonable regulation of the electronic structure of the catalyst can optimize its adsorption capacity for oxygen-containing intermediates, which is crucial for enhancing the catalytic performance of electrocatalysts [41]. EELS was used to analyze the local electronic interactions near the NCO/MO interface. Figure S13 displays the distribution of elements in the material. The results show that the lighter regions in Fig. 2c are concentrated with Co and Ni elements, which are identified as NiCo_2_O_4_, while MnO_2_ is distributed around them, which is consistent with the mapping results in Fig. 1h. The EELS scanning direction (red arrow in Fig. 2c) moves from MnO_2_ to NiCo_2_O_4_ and back to MnO_2_, and there are significant intensity changes and slight peak shifts on the Co L_3_ and Mn L_3_ edges, indicating strong electron interactions within the region [42, 43]. When NiCo_2_O_4_ and MnO_2_ are coupled to form a heterogeneous interface, the electronic structure of Co, Ni and Mn atoms is affected, which is manifested as the changes in the peak strength and positions of L_2,3_-edges on both sides of the interface (Fig. 2d-f). The results are consistent with the above XPS conclusions. According to the Mott–Schottky (M-S) analysis (Fig. S14) and UV–vis diffuse reflectance spectroscopy (Figs. S15 and 2 g), the possible energy band diagrams of NiCo_2_O_4_/MnO_2_, NiCo_2_O_4_, and MnO_2_ are displayed in Fig. 2h, i. Upon the construction of the NiCo_2_O_4_/MnO_2_ Mott–Schottky heterostructure, the electron interactions at the interface will spontaneously adjust the Fermi energy levels on both sides of the interface to reach an equilibrium state, which facilitates the charge transfer and alters the underlying reaction process, ultimately enhancing the catalytic activity of the material. The above results provide strong evidence for the successful construction of a heterogeneous interface between NiCo_2_O_4_ and MnO_2_.

### Analysis of Electrochemical Performances

**Fig. 3:**
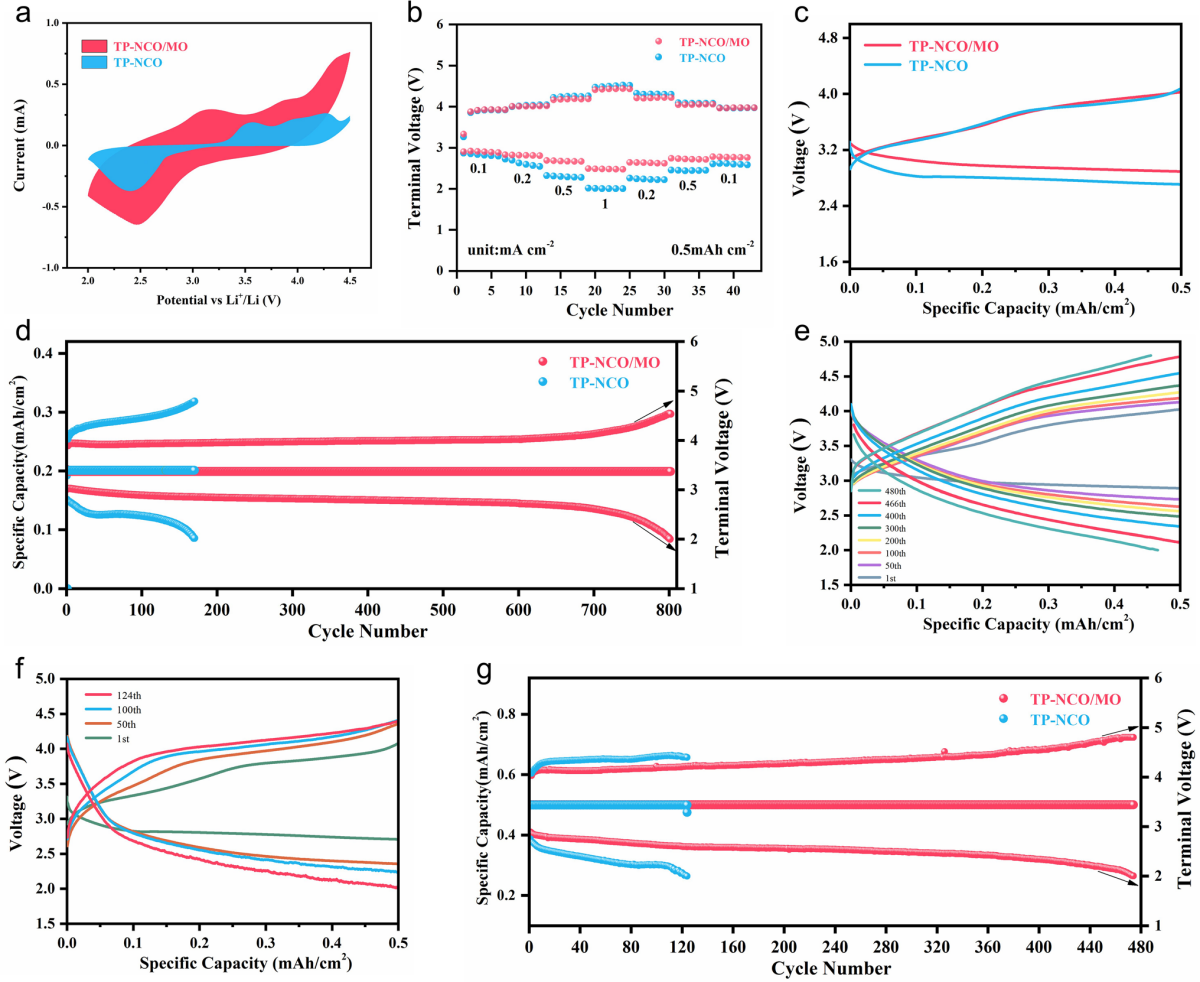
**a** CV curves of TP-NCO/MO and TP-NCO cathodes recorded at a sweep rate of 0.1 mV s^−1^. **b** Rate capability of TP-NCO/MO and TP-NCO cathodes at a fixed capacity of 0.5 mAh cm^−2^. **c** Voltage–capacity curves for the first cycle of TP-NCO/MO and TP-NCO cathodes at a current density of 0.5 mA cm^−2^. **d** Cycle performance of TP-NCO/MO and TP-NCO cathodes at 0.2 mA cm^−2^. The voltage–capacity curves of **e** TP-NCO/MO and **f** TP-NCO cathodes at a current density of 0.5 mA cm^−2^ with different number of cycles. **g** Variation of termination voltage as a function of cycle number of TP-NCO/MO and TP-NCO cathodes at a current density of 0.5 mA cm^−2^

To evaluate the electrocatalytic feasibility of TP-NCO/MO material, we directly used it as a free-standing air cathode for discharge and charge tests. Figure 3a displays the cyclic voltammetry (CV) curves of TP-NCO/MO and TP-NCO cathode materials in the voltage range of 2.0–4.5 V (vs. Li/Li^+^). The results show that the TP-NCO/MO cathode exhibits higher current density during the ORR/OER process and possesses more pronounced oxidation and reduction peaks [44, 45]. To evaluate the rate performance of TP-NCO/MO and TP-NCO catalyst materials, the current density was progressively raised from 0.1 to 1 mA cm^−2^ and then gradually lowered to 0.1 mA cm^−2^ under the limiting capacity of 0.5 mAh cm^−2^ (Fig. 3b). For TP-NCO/MO, the charging voltage only increases by only 0.05 V and the discharge termination voltage decreases by 0.13 V when the current density returns to 0.1 mA cm^−2^. However, for the TP-NCO cathode, when the current density restores to 0.1 mA cm^−2^, the charging voltage of the TP-NCO cathode increases by 0.46 V and the discharge termination voltage decreases by 0.28 V. The charge/discharge overpotential is also evaluated at 0.5 mA cm^−2^, and TP-NCO/MO and TP-NCO exhibit voltage gaps of 0.73 and 0.93 V, respectively (Fig. 3c). These results indicate that the NiCo_2_O_4_/MnO_2_ Mott–Schottky heterostructure significantly enhances ORR/OER kinetics, and the effect is mainly attributed to the dual role of MnO_2_ as an additional active site and electron rearrangement at the heterointerface. The changes in discharge/charge termination voltage during the cycle at 0.2 mA cm^−2^ are presented in Fig. 3d. For TP-NCO/MO, the termination voltage of charge and discharge is very stable during the first 700 cycles. In contrast, when TP-NCO was used as the cathode catalyst, the charging and discharging polarization gradually increased during the cycling process, and the discharge termination voltage dropped to a lower value after 170 cycles, which may be caused by the failure of timely decomposition and continuous accumulation of discharge products. Figure S18 illustrates the voltage–capacity curves of the two samples under the deep discharge at 0.2 mA cm^−2^. The discharge capacity of TP-NCO/MO was 6.02 mAh cm^−2^, while that of TP-NCO was only 4.34 mAh cm^−2^. It is well known that larger current densities and larger cutoff capacities in LOBs require higher electrocatalytic activity of the catalyst materials on the cathode side [46]. The cycling performance of the two materials was further assessed at a restricted capacity of 0.5 mAh cm^−2^ and a high current density of 0.5 mA cm^−2^. The discharge/charge curves of TP-NCO/MO and TP-NCO materials at different cycle times are shown in Fig. 3e, f. As shown in Fig. 3g, the TP-NCO/MO catalyst material can stably cycle for nearly 480 cycles at such a high current density, whereas the TP-NCO can only last for 124 cycles. Compared with other similar catalysts that have been reported, the materials prepared in this work have a significant advantage in terms of cycling life (Fig. S20 and Table S1).

### Reversibility Studies of Discharge Products

**Fig. 4:**
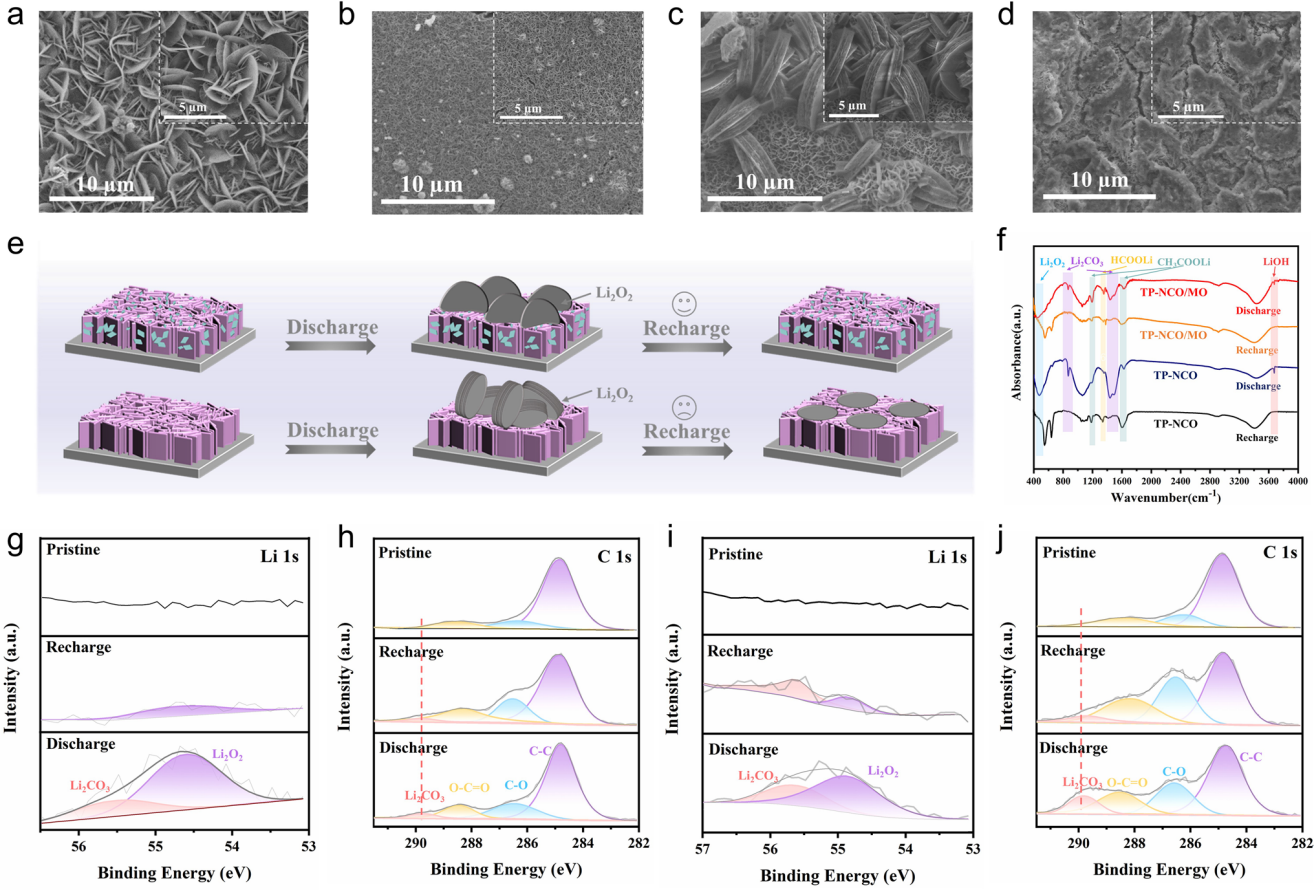
SEM images of TP-NCO/MO electrode at 0.2 mA cm^−2^ **a** after deep discharge and **b** recharge. SEM images of TP-NCO electrode at 0.2 mA cm^−2^ **c** after deep discharge and **d** recharge (further enlarged in the upper right corner). **e** Illustration of the discharge–recharge process of the TP-NCO/MO and TP-NCO cathodes. **f** FTIR spectra of TP-NCO/MO and TP-NCO electrodes in the discharge and recharge states. XPS fine spectra of **g** Li 1*s* and **h** C 1*s* in the initial, discharge and recharge states of TP-NCO/MO cathode at fixed capacity of 0.2 mAh cm^−2^ and current density of 0.2 mA cm^−2^ after 20 cycles. XPS fine spectra of **i** Li 1*s* and **j** C 1*s* under the same conditions for TP-NCO cathode

The morphology of the discharge products and the reversibility of the formation/decomposition were investigated using numerous characterization methods, such as SEM, XRD, XPS, FTIR, TEM, in situ EIS, and TOF–SIMs to understand the reaction mechanism within the TP-NCO/MO cathode. Figure 4a-d show the morphology of the TP-NCO/MO and TP-NCO surfaces in the full discharge and charge state at 0.2 mA cm^−2^, respectively. The insets provide enlarged views for better observation. For the TP-NCO/MO catalyst, after full discharge (Fig. 4a), it can be observed that a large "chip-like" discharge product is embedded within the nanosheet arrays, with a thickness of nearly 40 nm. The lower ends of the slice are in close contact with the NiCo_2_O_4_/MnO_2_ nanosheet arrays, which means that there are sufficient contact sites between the catalyst material and the discharge product, allowing for faster and more efficient decomposition, thus reducing overpotential and enhancing the rate performance of LOBs [28]. Adequate contact between the discharge product and the cathode is a crucial factor in achieving excellent electrochemical performance. However, the actual decomposition capability also depends on the interfacial properties between the electrolyte and the discharge product, as well as between the catalyst and the discharge product. Unsurprisingly, after recharge (Fig. 4b), the "chip-like" discharge products almost decompose and disappear, and the NiCo_2_O_4_/MnO_2_ nanosheet arrays are completely exposed. In sharp contrast, after the TP-NCO cathode material was completely discharged (Fig. 4c), "thousand-layer cookie-like " discharge products are observed at the top of the NiCo_2_O_4_ nanosheet arrays, with few contact sites with the electrode surface, which made subsequent fully reversible decomposition difficult, resulting in a large overpotential. It is noteworthy that the small MnO_2_ sheets distributed on the surface of NiCo_2_O_4_ nanosheets of TP-NCO/MO material can provide the initial nucleation site for the deposition of the discharge products, allowing the "chip-like" discharge products to be embedded and grown in the nanosheet arrays. After recharge (Fig. 4d), some residues of the "thousand-layer cookie-like" discharge products remain on top of the TP-NCO nanosheet arrays, implying poor reversibility. Based on the above results, a schematic diagram of the formation and decomposition of discharge products in TP-NCO/MO and TP-NCO cathodes can be presented (Fig. 4e). XRD test results of TP-NCO/MO at both discharge and recharge stages suggest that the formation and decomposition of Li_2_O_2_ are well reversible (Fig. S23) [28]. To further investigate the changes of all the discharge products of TP-NCO/MO and TP-NCO catalyst materials during discharge and recharge, the Fourier transform infrared (FTIR) spectra of the two catalysts were tested at 0.2 mA cm^−2^ at the full discharge and recharge stages (Fig. 4f), which provides strong evidence for the types and changes of discharge products after discharge and recharge. For TP-NCO/MO catalyst materials, Li_2_O_2_ (472 cm^−1^), Li_2_CO_3_ (860, 1437, and 1510 cm^−1^), HCOOLi (1360 cm^−1^), CH_3_COOLi (1197 and 1615 cm^−1^) and LiOH (3675 cm^−1^) were detected in the discharged state. After recharge, Li_2_O_2_, Li_2_CO_3_ and LiOH decomposed and disappeared, while HCOOLi and CH_3_COOLi showed little change. For TP-NCO catalyst materials, the same discharge products were detected. However, after recharge, the results showed that the by-products CH_3_COOLi and HCOOLi not only did not decompose, but also accumulated further. In contrast, TP-NCO/MO accumulated much less by-products during the cycling process, which was attributed to its lower charging voltage, reducing the occurrence of electrolyte decomposition and other side reactions caused by high potential [47]. In addition, the products of the discharge and recharge stages were further characterized by XPS (Fig. 4g-j). For the TP-NCO/MO cathode (Fig. 4g, h), the peaks fitted at 54.8 and 55.6 eV in the Li 1*s* fine spectrum at the discharge stage are related to Li_2_O_2_ and Li_2_CO_3_, respectively, and the corresponding peaks at 289.8 eV in the C 1*s* fine spectrum correspond to Li_2_CO_3_, which is almost completely decomposed after recharge. In contrast, in the TP-NCO cathode (Fig. 4i, j), both Li 1*s* and C 1*s* fine spectra were fitted to obtain a larger area of peaks for the by-product Li_2_CO_3_, which was not effectively decomposed after recharge.

**Fig. 5:**
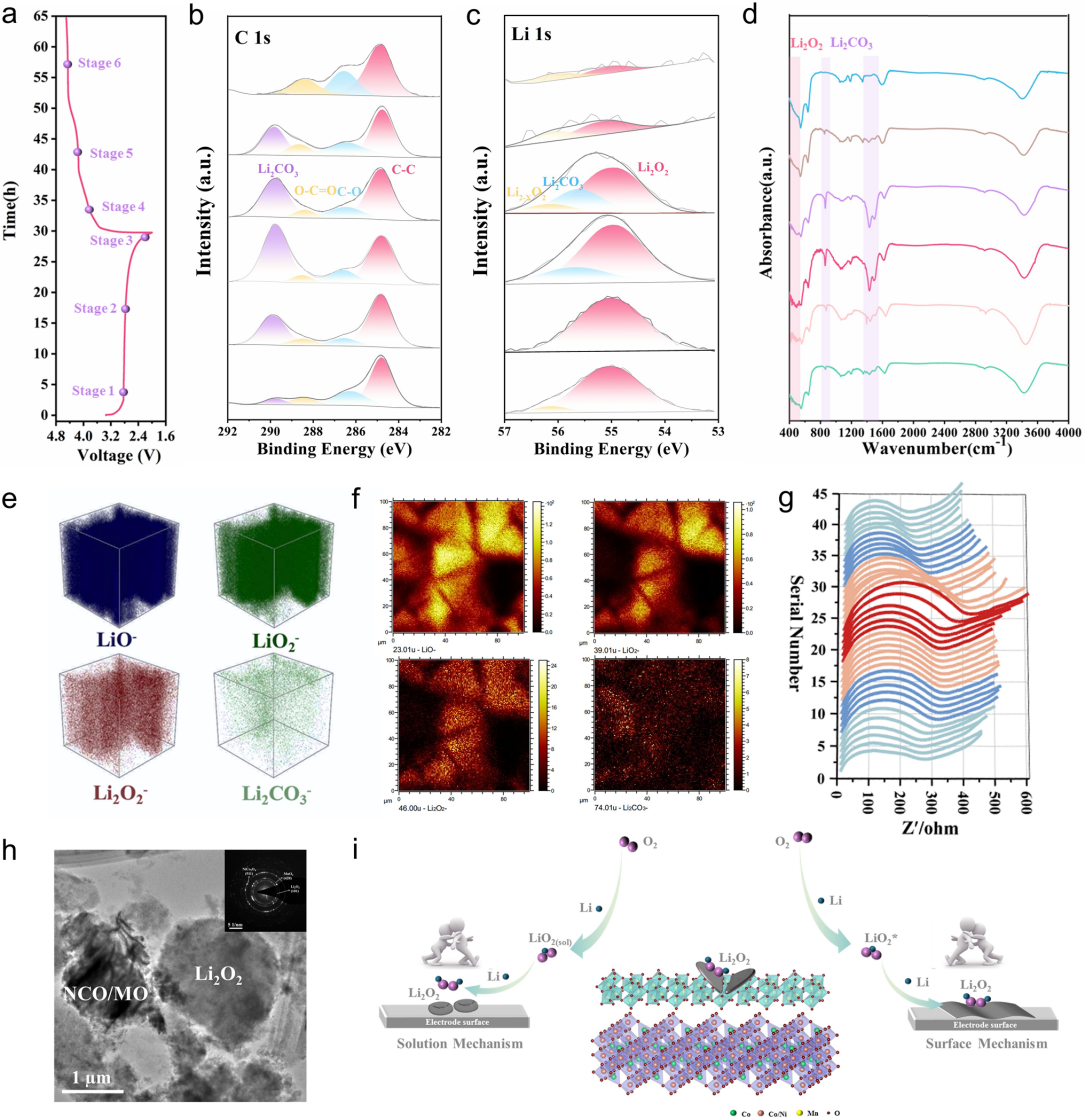
**a** Discharge/charge curve of TP-NCO/MO electrode at 0.2 mA cm^−2^. XPS fine spectra of **b** C 1*s* and **c** Li 1*s* and **d** FTIR spectra corresponding to stages (1 − 6). **e** 3D depth image and **f** the cross-sectional image of LiO^−^, LiO_2_^−^, Li_2_O_2_^−^, and Li_2_CO_3_^−^. **g** In situ EIS spectrum of TP-NCO/MO. **h** TEM of NCO/MO cathode after deep discharge at 0.2 mA cm^−2^ (SAED in insert). **i** Schematic reaction mechanism of TP-NCO/MO

The ex situ XPS and FTIR results of the discharge–recharge process further provide evidences for the high reversibility of the discharge products. Discharge and charge with unlimited capacity were performed at 0.2 mA cm^−2^, during which 6 voltages were intercepted for XPS and FTIR characterization in this process, as shown in Fig. 5a-d. The XPS C 1*s* fine spectrum results show that the peak area corresponding to Li_2_CO_3_ at 289.8 eV gradually increases during the discharge process and then gradually decreases after charging until it disappears at stage 6 (Fig. 5b). For the XPS Li 1*s* fine spectrum (Fig. 5c), in stage 1, the peaks at 55.1 and 56.1 eV correlate to Li_2_O_2_ and Li-deficient phase Li_2-x_O_2_, respectively. The formation/decomposition of Li_2_O_2_ can induce the generation of Li_2-x_O_2_ [48, 49]. Further discharge to stages 2 and 3 results in the formation of crystalline Li_2_O_2_ as the main product. In addition, a peak belonging to Li_2_CO_3_ appears at stage 3 and the FTIR characterization shows the same result (Fig. 5d). In the process of recharge, Gaussian fitted peaks of Li_2_O_2_ and the Li-deficient phase Li_2-x_O_2_ appeared at stages 4, 5, and 6. Differently, the peak area ratios of Li_2_O_2_ and Li_2-x_O_2_ gradually decrease with the recharging, indicating the gradual oxidation of Li_2_O_2_ to Li_2-x_O_2_ and subsequent decomposition. In addition, the peak corresponding to Li_2_CO_3_ remains visible in stage 4, but then disappears, suggesting that the by-products generated during discharge and recharge can also be decomposed in time to a certain extent, which is also confirmed by the corresponding FTIR characterization results.

To further analyze the composition and distribution of the discharge products, a TOF–SIMS analysis was performed on the TP-NCO/MO discharged electrode. LiO^−^, LiO_2_^−^, Li_2_O_2_^−^, and weak Li_2_CO_3_^−^ signals were detected in selected regions (Figs. S24 and 5e, f). The results demonstrate that the discharge product primarily consisted of non-stoichiometric Li_2-x_O_2_ [50], which is uniformly distributed throughout the three-dimensional space. Even under unlimited discharge conditions, the amount of by-product Li_2_CO_3_ remains low, which is consistent with the expected reaction that may occur during the discharge stage. The in situ EIS spectrum of TP-NCO/MO was used to monitor the evolution of electrochemical impedance during charging and discharging of LOBs (Fig. 5g). Discharge and charge were conducted for 2 h at 0.2 mA cm^−2^, and 40 sets of EIS spectra were recorded. The results of the equivalent circuit fitting of the representative 10 sets of data are shown in Table S4. In the initial state, two semicircles appear in the mid-frequency region of the EIS spectra. This may be due to the fact that the interface is not fully stabilized at the initial stage, resulting in the appearance of additional semicircles [51, 52]. During the subsequent discharge, it was replaced by a semicircle of increasing diameter, and the charge transfer impedance *R*_ct_ increased from 199.8 to 313 Ω, indicating the gradual accumulation of discharge products. At the end of charging, the discharge products are decomposed, and the charge transfer impedance *R*_ct_ gradually decreases to 208.7 Ω. After discharge, "chip-like" Li_2_O_2_ were further observed by TEM (Fig. 5h). Selective area electron diffraction (SAED) results confirmed the existence of NiCo_2_O_4_, MnO_2_, and Li_2_O_2_. According to the above experimental results, the possible formation mechanism of Li_2_O_2_ during the discharge processes of TP-NCO/MO is shown in Fig. 5i. Based on previous reports [24, 53], the morphology of Li_2_O_2_ formed during the discharge process is determined by the adsorption free energy on the electrode surface of the intermediate and the dissolution free energy of the electrolyte. It can be found that the TP-NCO/MO heterostructure materials obtained chip-like Li_2_O_2_ embedded nanosheet arrays under the competition of surface and solution mechanisms. This cross-embedded arrangement not only provides abundant contact sites with the electrode but also prevents passivation of the electrode.

### Theoretical Insights into Superior Performance

**Fig. 6:**
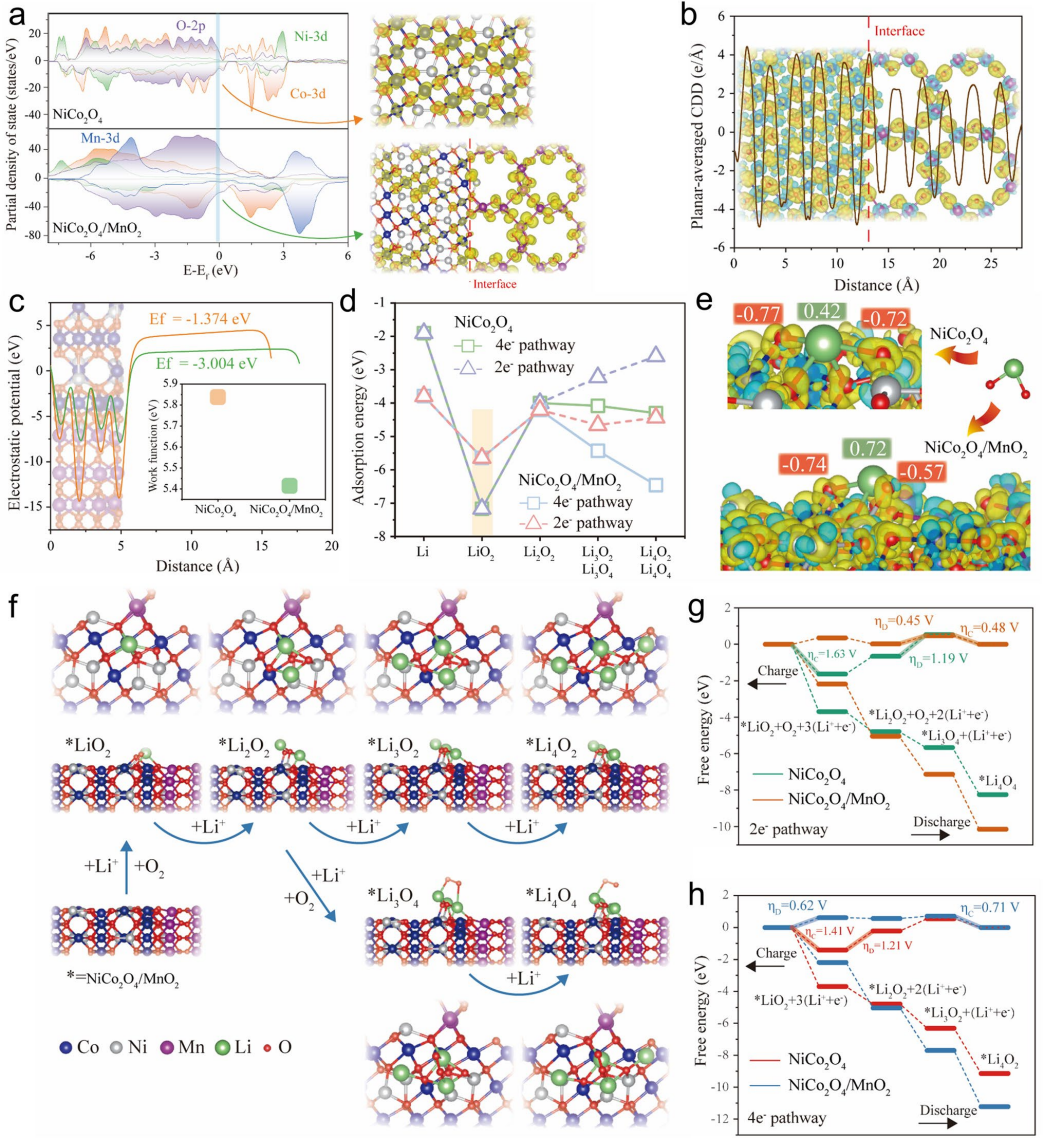
**a** Partial density of states of NiCo_2_O_4_ and NiCo_2_O_4_/MnO_2_, respectively. The diagrams are the charge densities near the Fermi energy level. **b** Planar-averaged charge density difference (CDD) across the z-direction for the NiCo_2_O_4_/MnO_2_ heterostructure. **c** Plot of the electrostatic potential along the NiCo_2_O_4_/MnO_2_ slab model. The inset is the work function of NiCo_2_O_4_ and NiCo_2_O_4_/MnO_2_. **d** Adsorption energy of the discharge intermediates for 4e^−^ pathway and 2e^−^ pathway, respectively. **e** CDD and Bader charge for the LiO_2_ adsorbed at NiCo_2_O_4_, NiCo_2_O_4_/MnO_2_. **f** Atomic configurations of the possible reaction paths of Li^+^ and O_2_ on the surface of NiCo_2_O_4_/MnO_2_. Free energy diagrams of the **g** 2e^−^ pathway and **h** 4e^−^ pathway for the discharge and charge process on the NiCo_2_O_4_ and NiCo_2_O_4_/MnO_2_

DFT calculations have been employed to unravel the underlying mechanisms through which heterostructures comprising NiCo_2_O_4_ and MnO_2_ can enhance the catalytic performance in LOBs. Detailed modeling specifics can be found in Fig. S28. In Fig. 6a, the partial density of states (PDOS) of NiCo_2_O_4_ and NiCo_2_O_4_/MnO_2_ have been computed, revealing that both systems exhibit metallic characteristics, with the density of states at the Fermi level (E_f_) primarily governed by O-2*p* and Co-3*d* orbitals. It is noteworthy that NiCo_2_O_4_/MnO_2_ exhibits a greater electron density in the vicinity of E_f_ compared to NiCo_2_O_4_. This increased electron density stems mainly from the contributions of Mn-3*d* and O-2*p* orbitals in MnO_2_. It is suggested that the assembly of NiCo_2_O_4_ and MnO_2_ heterostructures can significantly enhance the conductivity of the catalyst materials. Such enhancement is highly advantageous for promoting the three-phase reaction at the cathode-side interface. The illustration in Fig. 6a effectively portrays the charge density surrounding the Fermi level of NiCo_2_O_4_ and NiCo_2_O_4_/MnO_2_. By examining the image, it becomes evident that the density of states primarily originates from the Co and O atoms for NiCo_2_O_4_, while for NiCo_2_O_4_/MnO_2_, it is primarily contributed by Co, Mn, and O atoms at the surface. Figure 6b demonstrates the charge redistribution occurring at the NiCo_2_O_4_/MnO_2_ heterogeneous interface through the charge density difference (CDD). At the interface of NiCo_2_O_2_@MnO_2_ heterojunction, a series of interface interactions will occur due to the differences in crystal structure and electronic state of the two materials. Specifically, the Mn atoms in MnO_2_ tend to lose some electrons to form positively charged ions (Mn^3+^ or Mn^4+^). Meanwhile, the oxygen atoms on the surface of NiCo_2_O_2_ tend to gain these electrons, forming negatively charged ions (O^2−^). This charge redistribution leads to strong electrostatic interactions at the interface, resulting in a stable heterojunction structure. The exquisite lattice compatibility between NiCo_2_O_4_ and MnO_2_ gives rise to undulating and uninterrupted electric field variations at the interface. This phenomenon, coupled with the contrasting wave amplitudes exhibited by NiCo_2_O_4_ and MnO_2_, exerts a profound influence on managing the charge transfer process during the adsorption of intermediates while ensuring the remarkable stability of the interface by preserving its high level of structural integrity. The work function of both NiCo_2_O_4_ and NiCo_2_O_4_/MnO_2_ which reflects the rate of reaction kinetics of the surface was calculated as depicted in Fig. 6c. The values for NiCo_2_O_4_ and NiCo_2_O_4_/MnO_2_ are measured to be 5.84 and 5.42 eV, respectively. Remarkably, the lower work function of NiCo_2_O_4_/MnO_2_ suggests a higher level of reactivity at the heterogeneous interface formed by MnO_2_. This result aligns with the experimental observation that the catalytic performance of NiCo_2_O_4_/MnO_2_ heterogeneous structure surpasses that of its counterpart, the NiCo_2_O_4_ homogeneous structure.

The adsorption energies of discharge/charge intermediates under two-electron and four-electron reaction pathways for NiCo_2_O_4_/MnO_2_ and NiCo_2_O_4_ are calculated in Fig. 6d. Specifically, the adsorption energy of NiCo_2_O_4_/MnO_2_ and NiCo_2_O_4_ on Li and the intermediate LiO_2_ are consistent under the two pathways. Notably, the adsorption energy of NiCo_2_O_4_/MnO_2_ on Li is stronger than that of NiCo_2_O_4_, and intriguingly, the adsorption of NiCo_2_O_4_/MnO_2_ on the intermediate product LiO_2_ is weaker compared to NiCo_2_O_4_. The diagram of CDD and Bader charge of LiO_2_ adsorbed on the surface of NiCo_2_O_4_ and NiCo_2_O_4_/MnO_2_ in Fig. 6e demonstrate that Li loses more electrons, while O receives fewer electrons after the construction of the heterostructures, which is the reason behind the weaker adsorption of LiO_2_ on NiCo_2_O_4_/MnO_2_. More electrons lost by Li may be transferred to the substrate MnO_2_, as analyzed in Fig. 6b. The weak adsorption of the intermediate product LiO_2_ within an optimal range facilitates the swift conversion of the intermediate to the final product. Consequently, this accelerates the kinetics of discharge product formation. Furthermore, this appropriate adsorption of LiO_2_ allows the discharge product to strike a balance between surface-mediated and solution-mediated mechanisms. As a result, it assumes a chip-like morphology that is embedded, possesses high capacity, and offers more contact sites with the catalyst substrate, ultimately leading to highly reversible and high-capacity LOBs. Moreover, as adsorption progresses, NiCo_2_O_4_/MnO_2_ demonstrates stronger adsorption energy than NiCo_2_O_4_. Among these observations, the adsorption is particularly potent along the four-electron pathway. However, the adsorption energy of different intermediates within the four-electron pathway exhibits significant variation, thereby resulting in a larger overpotential. Further analysis on this matter will be explored in subsequent discussions. Figure 6f presents the schematic diagram depicting the reaction route at each step in the discharge process on the surface of NiCo_2_O_4_/MnO_2_. The corresponding redox free energies of NiCo_2_O_4_ and NiCo_2_O_4_/MnO_2_ following the four-electron pathway and the two-electron pathway at each step are exhibited in Fig. 6g, h. In the two-electron path (Fig. 6g), NiCo_2_O_4_ exhibits a maximum overpotential η_D_ of 1.19 V, whereas NiCo_2_O_4_/MnO_2_ demonstrates a significantly lower η_D_ of only 0.45 V. On the contrary, during the charge process, NiCo_2_O_4_ displays an overpotential η_C_ of 1.63 V, while NiCo_2_O_4_/MnO_2_ exhibits a much lower η_C_ of only 0.48 V. These observations indicate that NiCo_2_O_4_/MnO_2_ possesses a lower overpotential in the discharge/charge process, which is in alignment with the prior battery test results. Similar trends are highlighted in the four-electron path (Fig. 6h). The discharge and charge overpotentials η_D_/η_C_ of NiCo_2_O_4_ and NiCo_2_O_4_/MnO_2_ are 1.21/1.41 and 0.62/0.71 V, respectively, with both NiCo_2_O_4_/MnO_2_ exhibiting lower overpotentials. Moreover, our findings reveal that the discharge/charge overpotential of the four-electron path for the NiCo_2_O_4_/MnO_2_ catalyst is higher than that of the two-electron path, which concurs with the results depicted in Fig. 6d, further supporting the notion that the catalytic process is more favorable through the two-electron pathway, consistent with experimental results. These calculation results illustrate that constructing effective heterostructures is a crucial strategy in designing high-efficiency electrocatalysts for LOBs.

## Conclusions

In summary, self-supporting NiCo_2_O_4_/MnO_2_ with a Mott–Schottky heterogeneous structure was successfully prepared on titanium paper (TP-NCO/MO). The construction of the NCO/MO Mott–Schottky heterostructure triggers the interface disturbance and changes in the energy band structure to fundamentally optimize the adsorption of reaction intermediates, refine the morphology of the discharge products, and enhance the efficiency of the generation and dissolution of the discharge products. In addition, MnO_2_ serves as an effective active site in OER and ORR, and by providing additional reaction sites, MnO_2_ contributes to the acceleration of the reaction rate and the decrease of the overpotential, thus enhancing the catalytic activity. DFT calculations were used to understand how the NCO/MO heterostructures enhance the dynamics of ORR and OER in LOBs. The assembly of this heterostructure enhances the electrical conductivity of the catalyst materials, resulting in a more efficient three-phase reaction at the cathode interface. The lattice compatibility between NiCo_2_O_4_ and MnO_2_ creates undulating electric field variations at the interface, which, combined with their contrasting wave amplitudes, influences the charge transfer process during the adsorption of intermediates and maintains the structural integrity of the interface. As a result, TP-NCO/MO as a cathode catalyst for LOB exhibits excellent catalytic activity. This work regulates the morphology of discharge products by constructing Mott–Schottky heterostructures using a facile method, providing reference significance for designing efficient catalysts to optimize the adsorption of reaction intermediates.

## Supplementary Information

Below is the link to the electronic supplementary material.

Supplementary file1 (PDF 2069 kb)
