# Supplementary material for: Constructed Mott–Schottky Heterostructure Catalyst to Trigger Interface Disturbance and Manipulate Redox Kinetics in Li-O2 Battery
Source: Nanomicro Lett. 2024 Jul 29;16:258. doi: 10.1007/s40820-024-01476-4 (PMC11286616; doi:10.1007/s40820-024-01476-4)
Supplement: Supplementary file 1 — Supplementary file1 (PDF 2069 kb) [file 40820_2024_1476_MOESM1_ESM.pdf]

Supporting Information for

Constructed Mott–Schottky Heterostructure Catalyst to Trigger Interface Disturbance and Manipulate Redox Kinetics in Li-O_2 Battery

Yongji Xia[1], Le Wang[1], Guiyang Gao[1], Tianle Mao[1], Zhenjia Wang[1], Xuefeng Jin[1], Zheyu Hong[1], Jiajia Han[1,*], Dong-Liang Peng[1,*], Guanghui Yue[1,*]

[1]State Key Lab of Physical Chemistry of Solid Surface, Fujian Key Laboratory of Surface and Interface Engineering for High Performance Materials, College of Materials, Xiamen University, Xiamen 361005, P. R. China

*Corresponding authors. E-mail: jiajiahan@xmu.edu.cn (Jiajia Han); dlpeng@xmu.edu.cn (Dong-Liang Peng); yuegh@xmu.edu.cn (Guanghui Yue)

Supplementary Figures and Tables

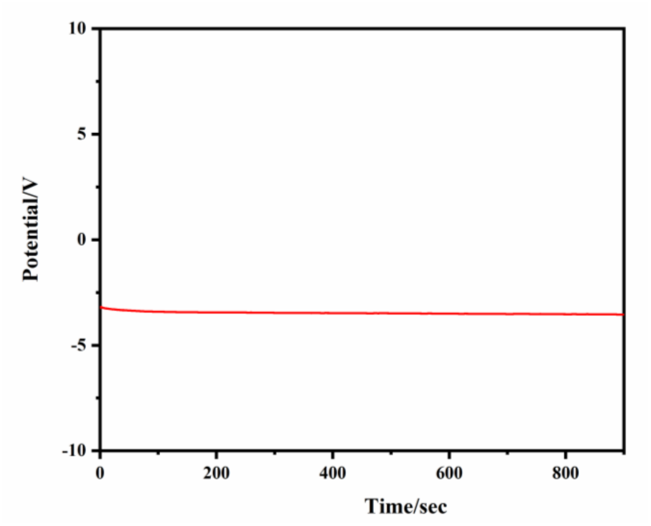

Fig. S1 Electrodeposition on Ti paper substance for preparing Ni-Co precursor

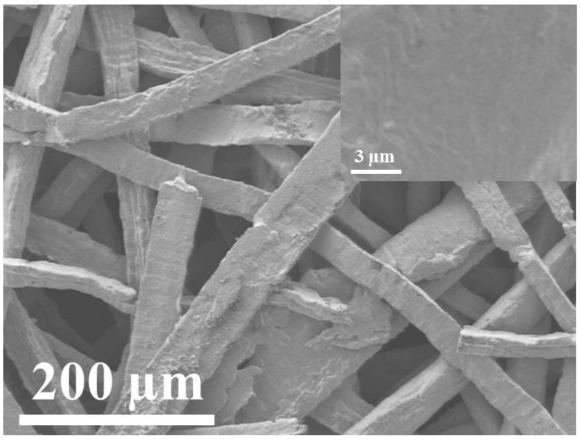

Fig. S2 SEM image of Ti paper (illustrated as a further enlarged image)

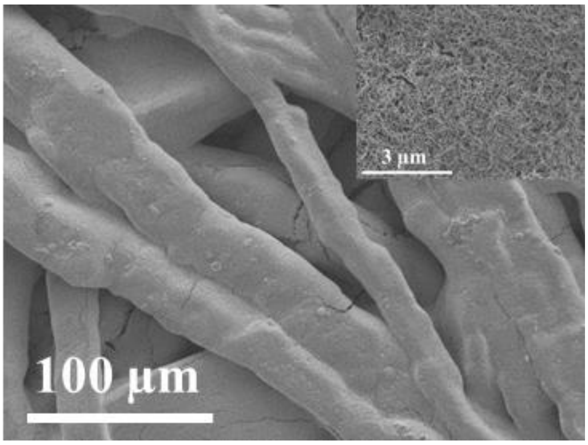

Fig. S3 SEM image of precursor (illustrated as a further enlarged image)

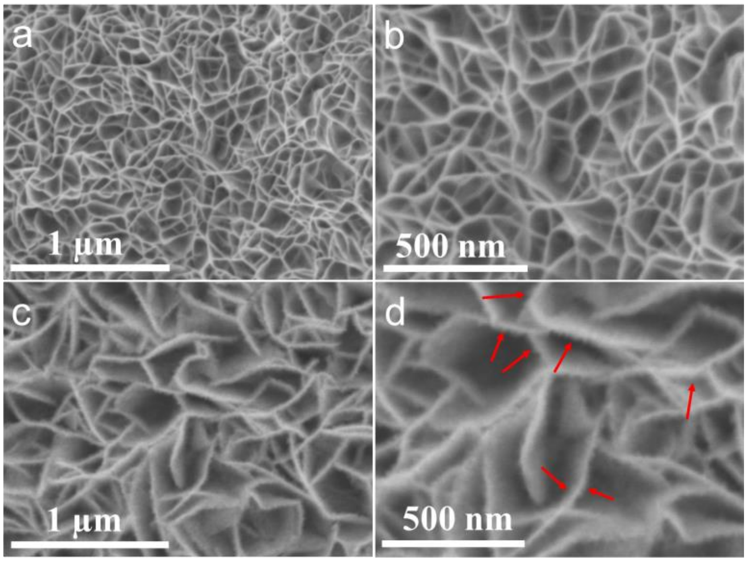

Fig. S4 SEM pattern of **a-b** TP-NCO and **c-d** TP-NCO/MO array

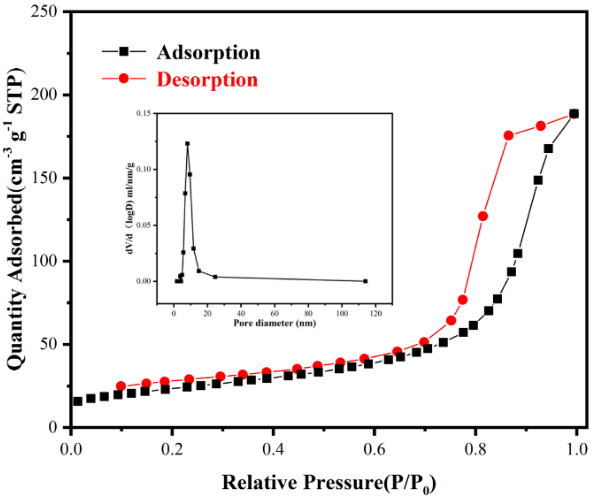

Fig. S5 N_2 absorption–desorption isotherm of TP-NCO. (the inset shows the pore-size distribution curve)

As shown in Fig. S2, the titanium paper consists of crossed titanium fibers of various sizes, and further magnification of the illustration reveals that the surface of the titanium fibers exhibits a smooth morphology. After the electrodeposition treatment, the cross distribution of titanium fibers remains apparent (Fig. S3), however, the enlarged inset reveals an interconnected porous network structure formed by the combination of vertically aligned nanosheets on titanium fiber surfaces. The three-dimensional mesh structure apparently provides a high specific surface area, enlarges the contact area of the catalyst with oxygen and electrolyte, accelerates the ORR and OER reactions at the three-phase interface, and provides adequate space for storage of discharge products. N_2 adsorption/desorption analysis confirms this (Fig. S5). The results show that $NiCo_2O_4$ nanosheets possess an extensive specific surface area as high as 83.3 m^2 g^{-1} with an average pore dimension of 13.9 nm.

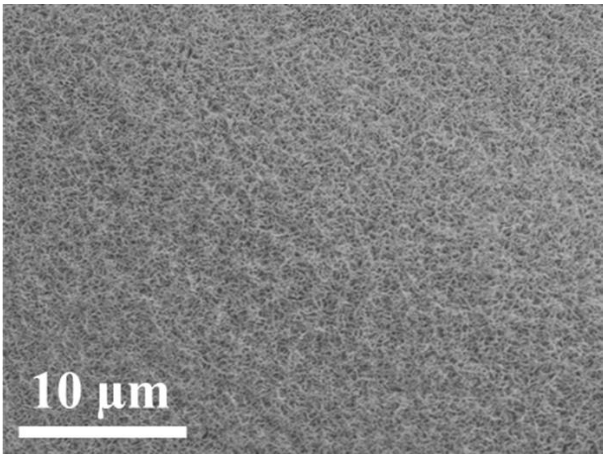

Fig. S6 SEM images of TP-NCO

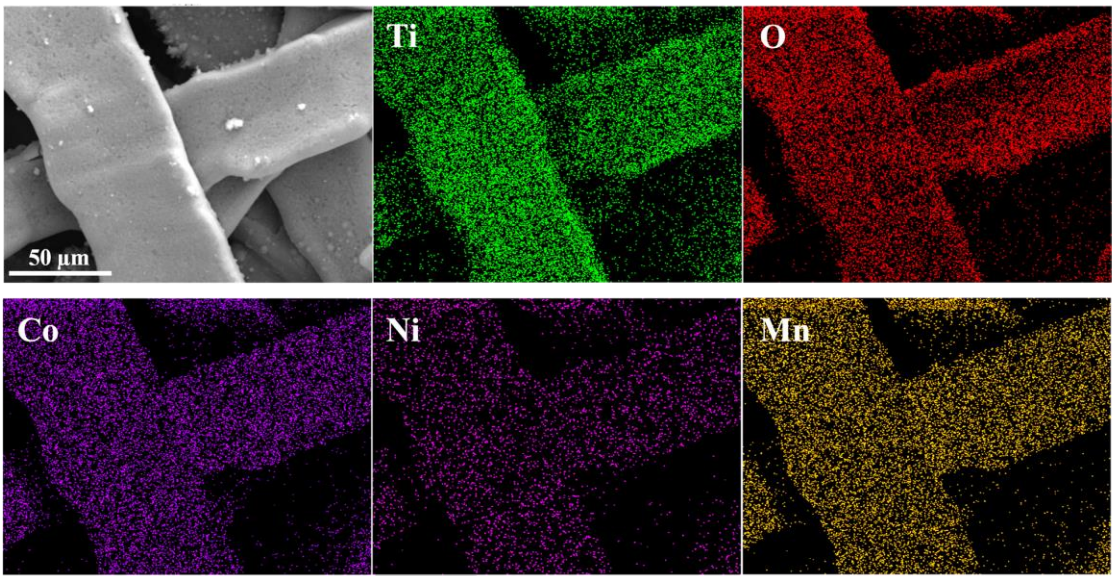

Fig. S7 EDS mapping pattern of TP-NCO/MO

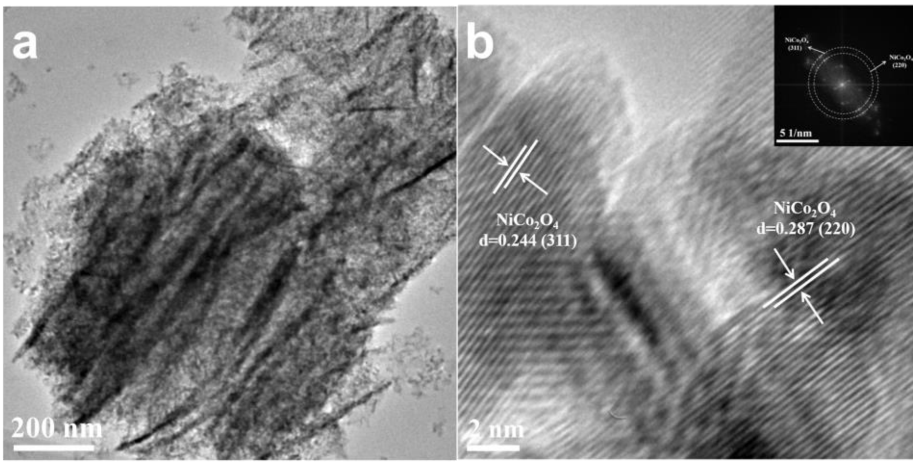

Fig. S8 a TEM pattern of $NiCo_2O_4$ array. **b** HRTEM pattern of $NiCo_2O_4$ array (inset shows the corresponding FFT pattern)

As a comparison, the TEM characterization of the TP-NCO catalyst material is shown in Fig. S8a-b. According to the HRTEM results, crystal plane spacing of 0.244 nm and 0.287 nm correlate to the (311) and (220) crystal planes of $NiCo_2O_4$, respectively.

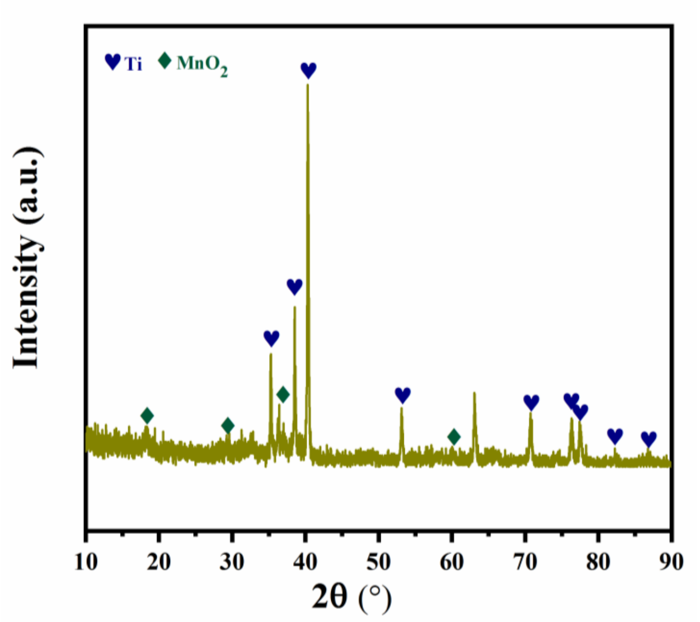

Fig. S9 XRD pattern of TP-MnO_2

For comparison, the XRD test was performed on the MnO_2 material grown on TP (Fig. S9), and the peaks at 18.0°, 28.9°, 36.7° and 60.1° corresponded to (200), (310), (400) and (521) crystal planes of MnO_2 (JCPDS No. 44-0141), which further validated the previous conclusions.

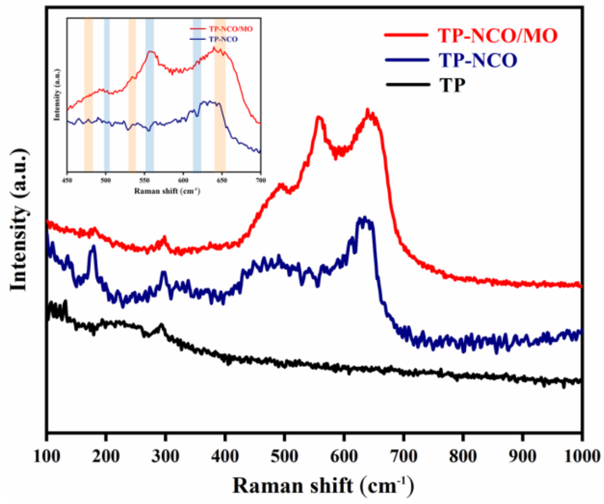

Fig. S10 Raman spectrum of TP, TP-NCO and TP-NCO/MO

Figure S10 displays the Raman spectra of TP-NCO/MO, TP-NCO, and TP materials, and the inset is a zoomed-in image. It can be seen that both TP-NCO/MO and TP-NCO materials have obvious peaks near 181, 475, 530, and 645 cm^{-1}, aligning with the F_{2g}, E_g, F_{2g} and A_{1g} vibration modes of $NiCo_2O_4$ (marked in orange) [S1]. Other peaks were detected near 500, 560, and 617 cm^{-1} in the TP-NCO/MO material, which are attributed to the symmetric tensile vibrations of the Mn-O bond within the MnO_2 lattice (marked in blue) [S2]. In addition, some small peaks that appear near 300 cm^{-1} are derived from the vibration of Ti-O bonds [S3, S4], which may be caused by an oxide layer on the TP surface after long-term exposure to air, as verified by the simultaneous occurrence of peaks in the TP-NCO/MO, TP-NCO and TP materials can verify this viewpoint (marked in green).

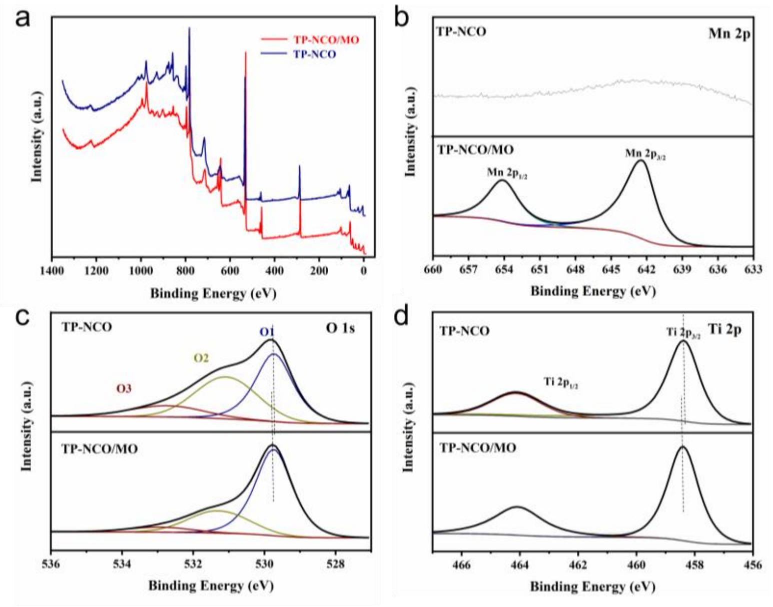

Fig. S11 a XPS survey spectra. High-resolution XPS spectrum of **b** Mn 2p, **c** O 1s and **d** Ti 2p of TP-NCO/MO and TP-NCO

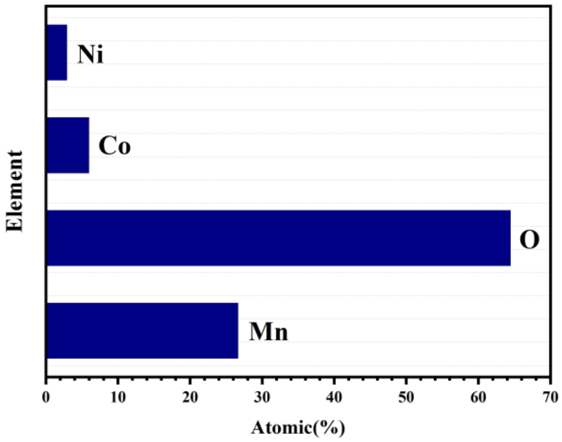

Fig. S12 The atomic ratio of Ni、Co、O and Mn

In the fine spectrum of Mn 2p (Fig. S11b), the characteristic peaks of TP-NCO/MO at 642.35 eV and 654.09 eV are ascribed to Mn 2p3/2 and Mn 2p1/2, respectively, with a peak separation of 11.74 eV. No obvious characteristic peaks were detected in TP-NCO, further confirming the existence of MnO_2 in TP-NCO/MO. Fig. S11c shows the high-resolution spectra of O 1s. For TP-NCO, the peaks at 529.72, 531.07, and 532.72 eV are related to lattice oxygen produced by metal oxides, surface oxygen vacancies (O_{vs}) and surface adsorbed hydroxides, respectively. However, these corresponding peaks appear at 529.75, 531.27, and 532.94 eV in TP-NCO/MO, with a slight positive shift. In addition, the proportions of lattice oxygen produced by metal oxides and O_{vs} in TP-NCO/MO materials were 66.7% and 25.8%, respectively, while the corresponding proportions in TP-NCO materials were 44.0% and 41.0%, which was attributed to the increase in the proportion of metal oxides after MnO_2 growth on TP-NCO, and part of the oxygen vacancies were refilled [S5]. A moderate amount of oxygen vacancies can generate defect states in the band gap, allowing for easier electron excitation and enhanced charge transfer, resulting in higher OER/ORR activity [S6]. In the fine spectrum of Ti 2p (Fig. S11d), the peaks at 458.37 and 464.12 eV correlate to Ti 2p3/2 and Ti 2p1/2 of Ti^{4+}, which originate from the oxide layer on the TP surface, in agreement with the results of Raman analysis. After the growth of MnO_2, both peaks are shifted forward by about 0.1 eV, possibly due to the formation of Ti-O-Mn bonds between the MnO_2 and TiO_2 interfaces [S5, S7].

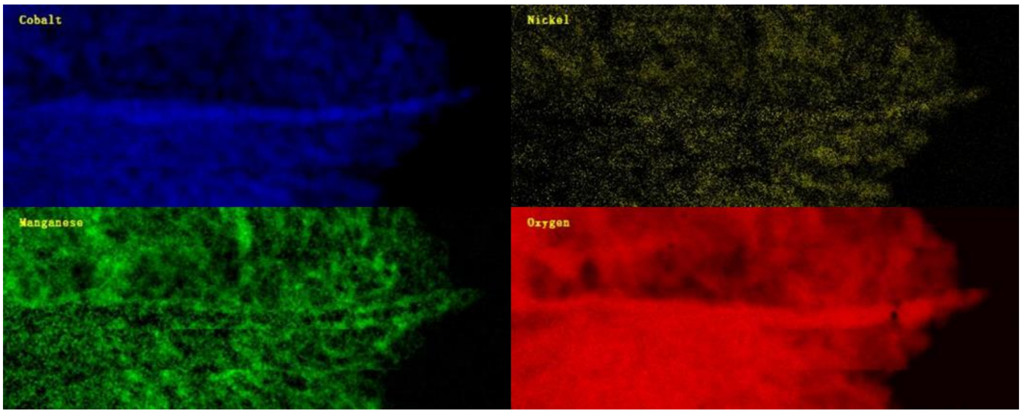

Fig. S13 Element mapping pattern of NCO/MO

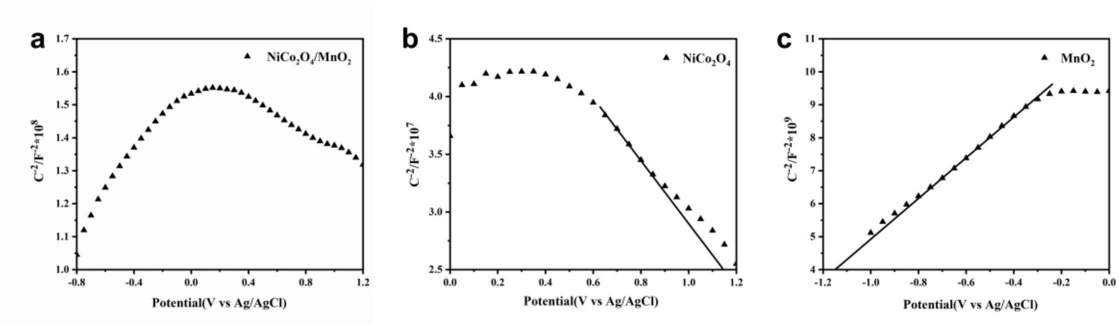

Fig. S14 Mott-Schottky plots of **a** $NiCo_2O_4/MnO_2$ **b** $NiCo_2O_4$ and **c** MnO_2

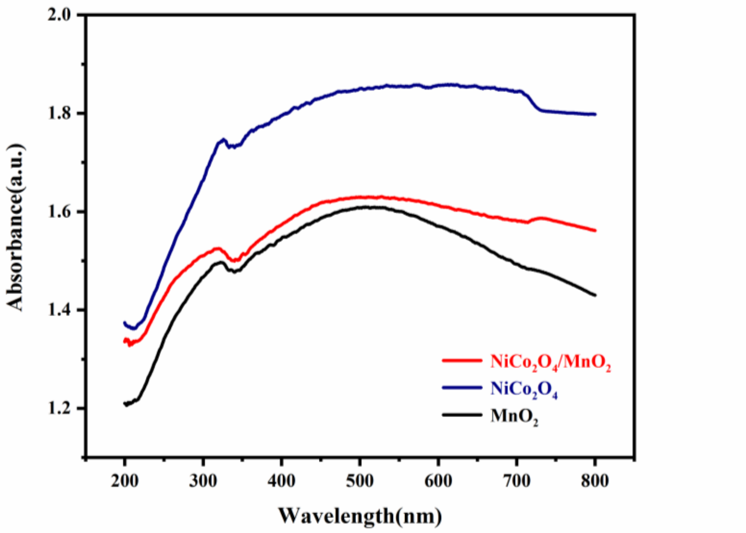

Fig. S15 UV–vis diffuse reflectance spectra of samples

Mott-Schottky (M-S) analysis was carried out on three materials, $NiCo_2O_4/MnO_2$, $NiCo_2O_4$ and MnO_2 (Fig. S14a-c), respectively. $NiCo_2O_4$ shows a negative slope recognized as a p-type semiconductor, while MnO_2 shows a positive slope recognized as an n-type semiconductor, and an "inverted V" curve is observed in the construction of the $NiCo_2O_4/MnO_2$ heterostructure, which confirms the formation of p-n junctions

[S8]. In addition, the M-S results allow the determination of the material flat-band potential values, where the valence-band (E_v) potential of p-type semiconductors and the conduction-band (E_c) potential of n-type semiconductors are close to the flat-band potentials, with a difference of 0.1-0.3 eV. As shown in Fig. S13b-c, the E_v of $NiCo_2O_4$ and the E_c of MnO_2 are 1.24 eV (1.44 eV vs. NHE) and -0.85 eV (-0.65 eV vs. NHE), respectively. The forbidden bandwidths of the three materials were calculated through UV-vis diffuse reflectance spectroscopy (Fig. S15), and Fig. 2g shows the bandgap derived according to the Kubelka-Monk theorem, which shows that the E_g of $NiCo_2O_4/MnO_2$, $NiCo_2O_4$, and MnO_2 are 1.15, 1.25, and 1.34 eV, respectively. According to these values, the E_c of $NiCo_2O_4$ and the E_v of MnO_2 were determined to be -0.01 and 0.49 eV, respectively.

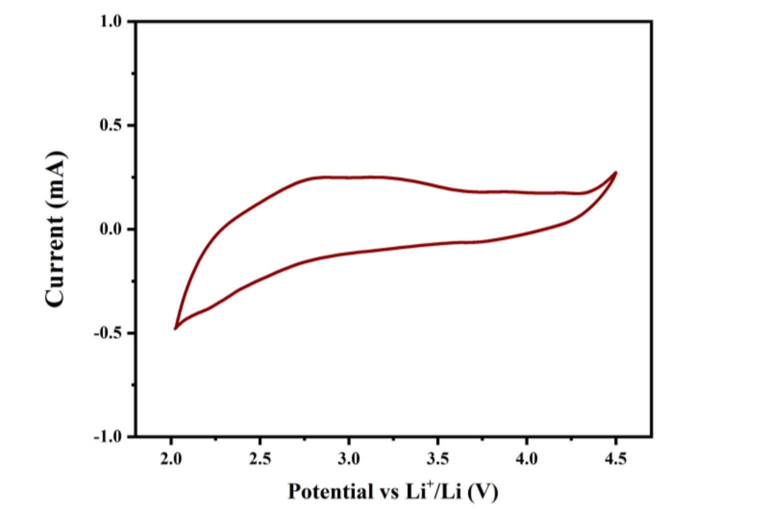

Fig. S16 CV curves of TP-NCO/MO cathodes measured at a sweep rate of 0.1mv s^{-1} in Ar atmosphere

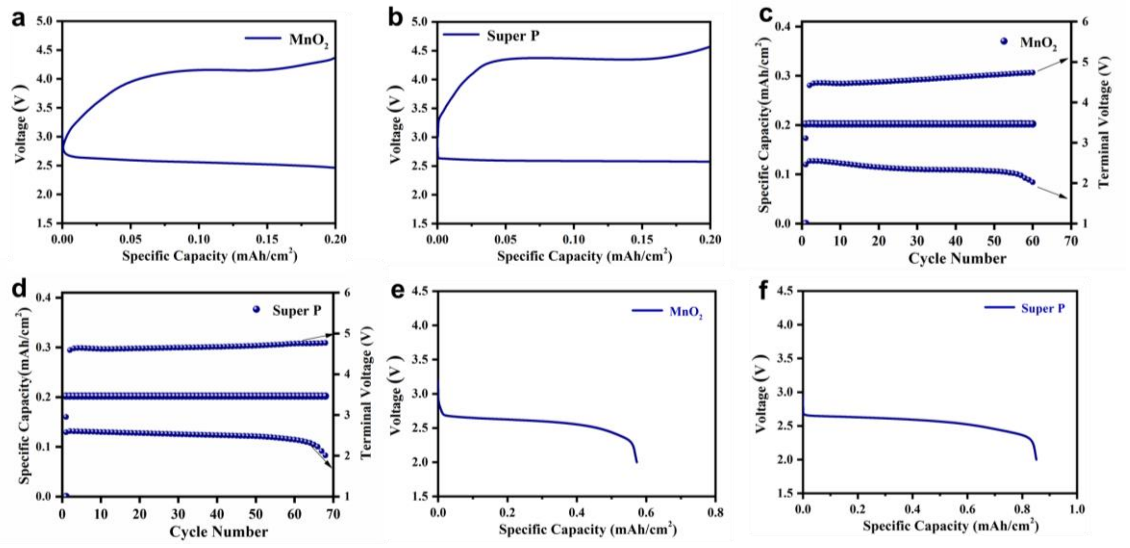

Fig. S17 The voltage-capacity curves for the first cycle at fixed capacity of 0.2 mAh cm^{-2} and current density of 0.2 mA cm^{-2} for **a** MnO_2 and **b** Super P cathode. The

variation of termination voltage as a function of cycle number at the fixed capacity of 0.2 mAh cm^{-2} and current density of 0.2 mA cm^{-2} for **c** MnO_2 and **d** Super P cathode. The voltage-capacity curves of deep discharge at 0.2 mA cm^{-2} for **e** MnO_2 and **f** Super P cathode

For comparison, MnO_2 and Super P catalyst materials loaded on carbon paper were measured under test conditions with a current density of 0.2 mA cm^{-2} and a cut-off capacity of 0.2 mAh cm^{-2}. Figure S17a-d displays that the charging/discharging gaps of MnO_2 and Super P are as high as 1.59 and 1.78 V d at the first cycle, with a cycle life of only 60 and 68 cycles, respectively. Meanwhile, the MnO_2 and Super P catalyst materials were deeply discharged at 0.2 mA cm^{-2} (Fig. S17e-f), their discharge capacities were only 0.57 mAh cm^{-2} and 0.85 mAh cm^{-2}, respectively, which were far inferior to TP-NCO/MO catalyst. The catalytic performance of the MnO_2 was poorer than that of the Super P due to the bad conductivity and slow reaction kinetics of the individually synthesized MnO_2.

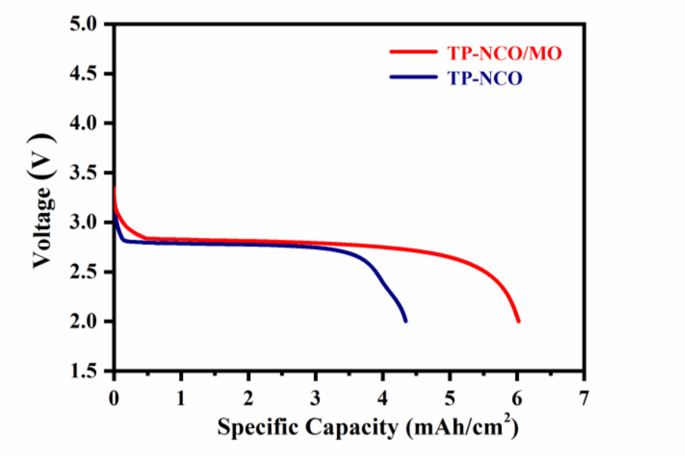

Fig. S18 Voltage-capacity curves of deep discharge at a current density of 0.2 mA cm^{-2} of TP-NCO/MO and TP-NCO cathodes

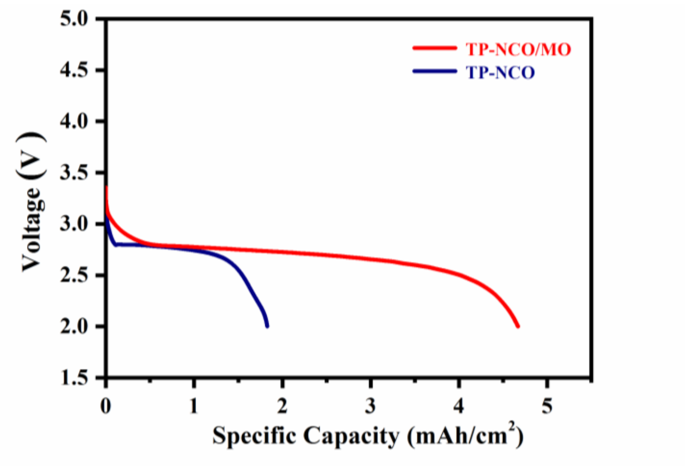

Fig. S19 Voltage-capacity curves of deep discharge at a current density of 0.5 mA cm^{-2} of TP-NCO/MO and TP-NCO cathodes

The unrestricted discharge capacity was also evaluated at 0.5 mA cm^{-2} (Fig. S19), and the results showed that the discharge capacities of TP-NCO/MO and TP-NCO catalyst materials were 4.67 and 1.82 mAh cm^{-2}, respectively, which indicated that TP-NCO/MO catalyst exhibited a higher discharge capacity than TP-NCO at different current densities.

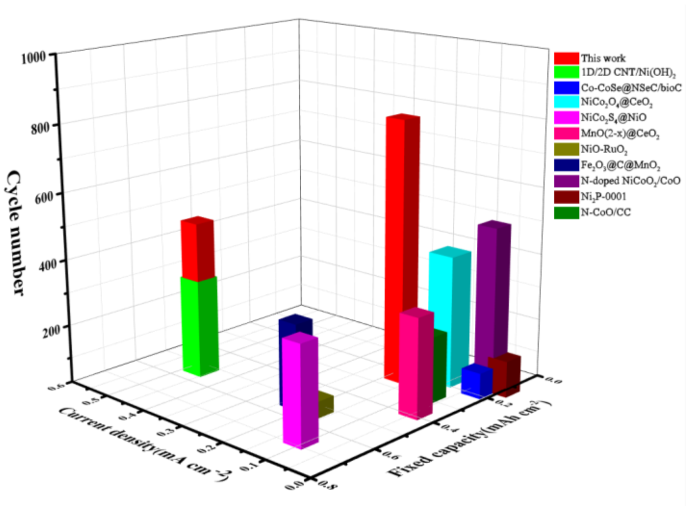

Fig. S20 The comparison of cycle performances in LOBs with different catalysts

Table S1 The comparison of cycle performances in LOBs with different catalysts

Catalyst	Current density (mA cm^{-2})/Fixed capacity (mAh cm^{-2})	Cycle number	References
1D/2D CNT/$Ni(OH)_2$	0.5/0.5	300	[S9]
Co-CoSe@NSeC/bioC	0.05/0.15	78	[S10]
$NiCo_2O_4$@CeO_2	0.13/0.13	400	[S11]
$NiCo_2S_4$@NiO	0.13/0.65	300	[S12]
MnO_{2-x}@CeO_2	0.07/0.35	302	[S13]
NiO-RuO_2	0.2/0.5	50	[S14]
Fe_2O_3@C@MnO_2	0.25/0.5	260	[S15]
N-doped $NiCoO_2$/CoO	0.05/0.1	500	[S16]
Ni_2P-0001	0.02/0.1	110	[S17]
N-CoO/CC	0.1/0.25	200	[S18]
This work	0.2/0.2<br>0.5/0.5	800<br>480	\

Table S2 The comparison of electrochemical performances with different catalysts

Catalyst (Battery Types)	Voltage gap (V)	Running time (h)	Discharge specific capacity	References
Pd/CNT ($Li-O_2/CO_2$)	1.68 V (500 mA g^{-1}-1000 mAh g^{-1})	1430 (500 mA g^{-1}-500 mAh g^{-1})	6628 mAh g^{-1} (500 mA g^{-1})	[S19]
Ir/AP-POP ($Li-O_2$)	0.03 V (0.04 mA cm^{-2} -0.12 mAh cm^{-2})	700 (0.04 mA cm^{-2} -0.12 mAh cm^{-2})	12.8 mAh (0.04 mA cm^{-2})	[S20]
Mo_3N_2 ($Li-CO_2$)	0.64 V (10 μA cm^{-2}-100μAh cm^{-2})	910 (20μA cm^{-2}-100 μA h cm^{-2})	8.361 mA h cm^{-2} (20 μA cm^{-2})	[S21]
CFB@NCNT-Mo_2N ($Li-CO_2$)	1.08V (10μA cm^{-1}-50μAh cm^{-1})	675 (40μA cm^{-2}-50μA cm^{-2})	5586.0μAh cm^{-1} (10 μA cm^{-1})	[S22]
BTO-CNT ($Li-O_2$)	0.65V (100mA g^{-1}-900 mAh g^{-1})	2000 (200 mA g^{-1}-500 mAh g^{-1})	18438 mAh g^{-1} (200 mA g^{-1})	[S23]
NiCoFeO@NF ($Li-O_2$)	0.2V (100mA g^{-1}-1000 mAh g^{-1})	790 (500 mA g^{-1}-1000 mAh g^{-1})	16727 mAh g^{-1} (500 mA g^{-1})	[S24]
Co/Fe@NC ($Li-O_2$)	1.003V (250mA g^{-1}-500 mAh g^{-1})	1000 (500mA g^{-1}-1000mAh g^{-1})	17326 mAh g^{-1} (125 mA g^{-1})	[S25]
Ni-HTP ($Li-O_2$)	0.88V (500mA g^{-1}-1000 mAh g^{-1})	800 (500mA g^{-1}-500mAh g^{-1})	15080 mAh g^{-1} (500 mA g^{-1})	[S26]
This work ($Li-O_2$)	0.73V (0.5 mA cm^{-2} -0.5 mAh cm^{-2})	1600 (0.2 mA cm^{-2} -0.2 mAh cm^{-2})	6.02 mAh cm^{-2} (0.2 mA cm^{-2})	\

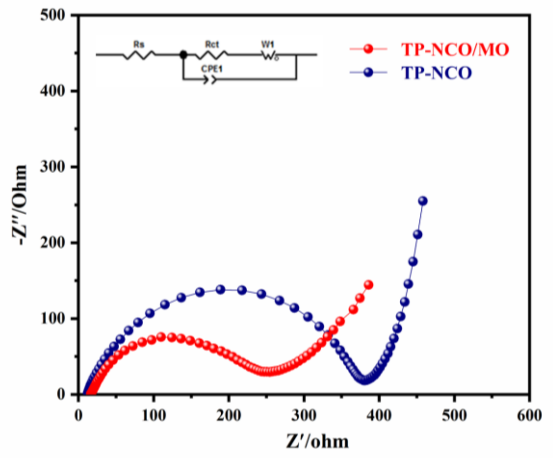

Fig. S21 EIS curves of TP-NCO/MO and TP-NCO cathodes

Table S3 Equivalent circuit parameters of the prepared samples

**Samples**	**Resistance (Ω)**	
	R_s	R_{ct}
**TP-NCO/MO**	17.34	186
**TP-NCO**	11.87	368.3

The electrochemical impedance spectra (EIS) of TP-NCO/MO and TP-NCO cathode materials were tested (Fig. S21), and the inset of Fig. S21 and Table S3 display the corresponding equivalent circuit diagrams and fitting results. The results show that the TP-NCO/MO cathode has a much lower charge transfer resistance (R_{ct}) value than TP-NCO, indicating better interfacial contact and faster charge transfer, thus facilitating the interfacial three-phase reactions and avoiding to some extent a series of problems due to the slow kinetics. The decrease in R_{ct} value proves that the construction of $NiCo_2O_4/MnO_2$ Mott-Schottky heterostructures improves the electrical conductivity and charge transfer ability, which is another advantage of this heterostructure.

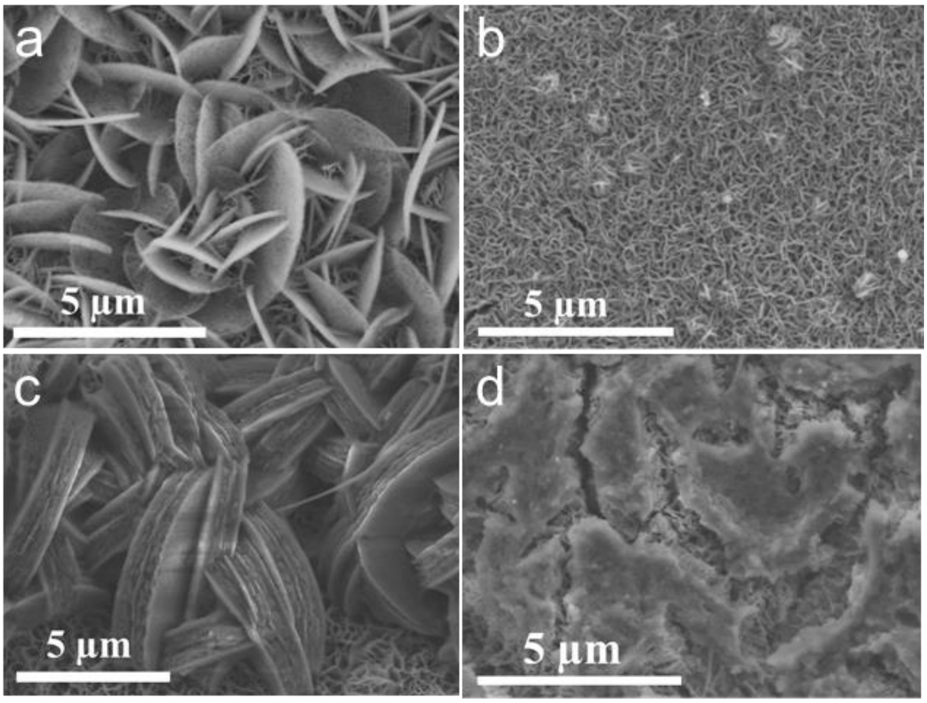

Fig. S22 Further enlarged SEM images of TP-NCO/MO electrode at 0.2 mA cm^{-2} **a** after deep discharge and **b** recharge. SEM images of TP-NCO electrode at 0.2 mA cm^{-2} **c** after deep discharge and **d** recharge

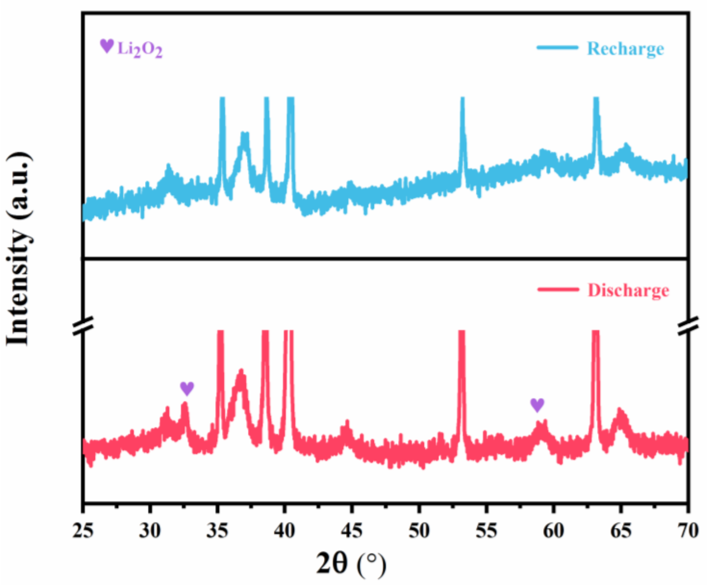

Fig. S23 XRD in the discharge and recharge states of TP-NCO/MO cathode

In the discharge state, the peaks identified at 32.8° and 58.6° correlate to the (100) and (110) crystal plane of Li_2O_2 (PDF#09-0355), respectively, and the peaks disappear after recharge.

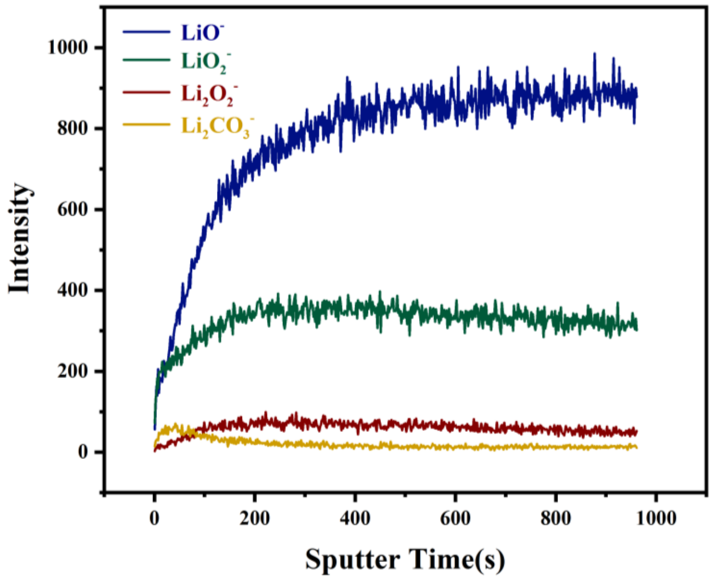

Fig. S24 The intensity evolution of LiO^-, LiO_2^-, $Li_2O_2^-$, and $Li_2CO_3^-$species as a function of the sputtering time

Table S4 Equivalent circuit parameters of TP-NCO/MO cathode in different states

Serial Number	Resistance (Ω)		
	R_1	R_2	R_3
1	9.4	87.2	199.8
5	11.0	/	246.9
10	12.2	/	308.9
15	13.5	/	310.0
20	15.0	/	313
21	15.4	/	318
25	17.8	/	392.6
30	17.4	/	271.9
35	18	/	238.1
40	19.8	/	208.7

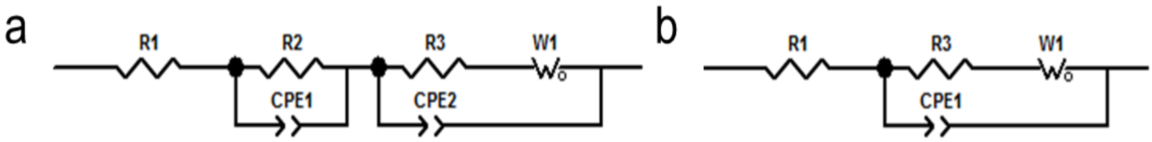

Fig. S25 Equivalent circuit models of **a** Serial Number 1 and **b** Serial Number 5-40

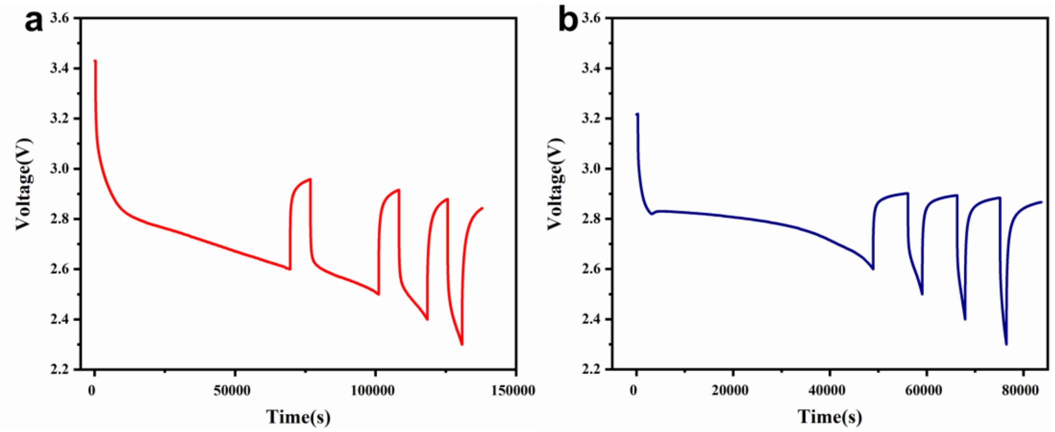

Fig. S26 GITT curves of **a** TP-NCO/MO and **b** TP-NCO

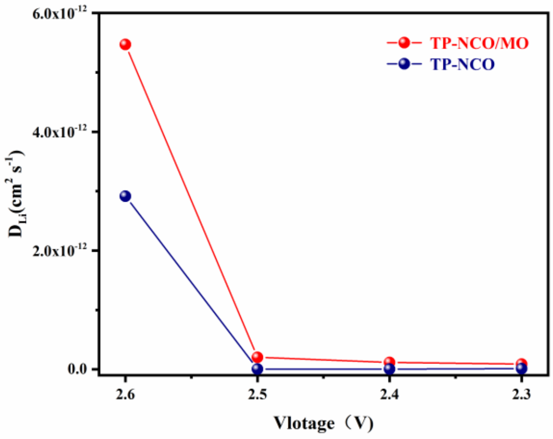

Fig. S27 Lithium diffusion rate of TP-NCO/MO and TP-NCO under different depths of discharge

The discharge settings of GITT were discharged to 2.6, 2.5, 2.4, and 2.3 V at a current density of 0.2 mA cm^{-2} with a relaxation time of 2 h. The lithium-ion diffusion coefficient (D_{Li}) was calculated by using the following equation:

$$D=4(\pi\tau)^{-1}(n_m V_M/S)^{2}(\Delta Es/\Delta Et)^2$$

where τ is the relaxation time, n_m is the number of moles, V_M is the molar volume, S is the electrode area, ΔE_s is the pulse-induced potential change, and ΔE_t is the potential change at the depth of discharge.

The D_{Li} for TP-NCO/MO calculated from Fick's second law equation at 2.6, 2.5, 2.4, and 2.3 V discharge depths are 5.46799× 10^{-12}, 2.01399× 10^{-13}, 1.15983× 10^{-13}, and 8.82813× 10^{-14} cm^2 s^{-1}, respectively. It is faster than 2.91628× 10^{-12}, 5.07459× 10^{-15}, 4.79301× 10^{-15}, and 1.11555× 10^{-14} cm^2 s^{-1} of TP-NCO (Figs. S26 and S27). Additionally, both TP-NCO/MO and TP-NCO exhibit gradually smaller D_{Li} values with the increase of Li_2O_2 content.

According to previous experience [S27], the galvanostatic intermittent titration technique (GITT) was able to assess the formation rate of Li_2O_2. The larger Li^+ diffusion coefficients (D_{Li}) further confirm that the TP-NCO/MO catalyst material exhibits faster reaction kinetics and superior catalytic performance (Figs. S26 and S27).

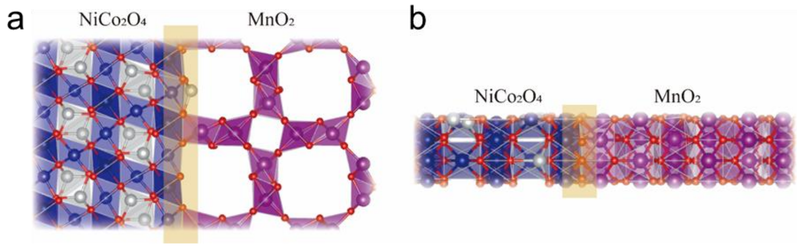

Fig. S28 a Top and **b** side views of the $NiCo_2O_4/MnO_2$ heterostructure model for DFT calculation

Supplementary References

[S1] S. Karmakar, R. Boddhula, B. Sahoo, B. Raviteja, D. Behera, Electrochemical performance of heterogeneous, mesopores and non-centrosymmetric core@shell $NiCO_2O_4$@MnO_2 nanocomposites and its MWCNT blended complex for supercapacitor applications. J. Solid State Chem. **280**, 121013 (2019). https://doi.org/10.1016/j.jssc.2019.121013

[S2] L. I. Li, H. U. Zhong-Ai, Y. Yu-Ying, W. U. Hong-Ying, C. U. I. Lu-Juan, Synthesis of a MnO_2/$NiCO_2O_4$ composite by electrostatic self-assembly and its electrochemical performance. Acta Phys.-Chim. Sin. **30**(5), 899-907 (2014). https://doi.org/10.3866/pku.Whxb201403261

[S3] M. Cieslak, D. Puchowicz, I. Kaminska, SEM/EDS and Raman micro-spectroscopy examination of titanium-modified polypropylene fibres. Fibres Text East Eur. **22**(3), 47-53 (2014). https://api.semanticscholar.org/CorpusID:67773652

[S4] U. Balachandran, N. G. Eror, Raman-spectra of titanium-dioxide. J Solid State Chem. **42**(3), 276-282 (1982). https://doi.org/10.1016/0022-4596(82)90006-8

[S5] X. Cheng, G. Dong, Y. Zhang, C. Feng, Y. Bi, Dual-bonding interactions between MnO_2 cocatalyst and TiO_2 photoanodes for efficient solar water splitting. Appl. Catal. B: Environ. **267**, 118723 (2020). https://doi.org/10.1016/j.apcatb.2020.118723

[S6] T. Hu, Z. Jiang, Z. Fu, Z.-J. Jiang, A NiFe/$NiSe_2$ heterojunction bifunctional catalyst rich in oxygen vacancies introduced using dielectric barrier discharge plasma for liquid and flexible all-solid-state rechargeable Zn–air batteries. J. Mater. Chem. A. **10**(16), 8739-8750 (2022). https://doi.org/10.1039/d2ta00949h

[S7] I. Iatsunskyi, M. Kempiński, G. Nowaczyk, M. Jancelewicz, M. Pavlenko et al., Structural and xps studies of PSI/TiO_2 nanocomposites prepared by ald and ag-assisted chemical etching. Appl. Surf. Sci. **347**, 777-783 (2015). https://doi.org/10.1016/j.apsusc.2015.04.172

[S8] J. Zheng, Z. Lei, Incorporation of coo nanoparticles in 3d marigold flower-like hierarchical architecture mnco2o4 for highly boosting solar light photo-oxidation and reduction ability. Appl. Catal. B: Environ. **237**, 1-8 (2018). https://doi.org/10.1016/j.apcatb.2018.05.060

[S9] B. Zhao, Y. Wu, L. Han, Z. Xia, Q. Wang et al., Collective, bifunctional 1d CNT/2D tmoh hybrid sponge as high-capacity and long-cycle Li-O_2 cathode. Energy Storage Mater. **50**, 344-354 (2022). https://doi.org/10.1016/j.ensm.2022.05.029

[S10] H. G. Liang, L. H. Jia, F. Chen, S. Y. Jing, P. Tsiakaras, A novel efficient

electrocatalyst for oxygen reduction and oxygen evolution reaction in Li-O_2 batteries: Co/cose embedded n, se co-doped carbon. Appl. Catal. B: Environ. **317**, 10 (2022). https://doi.org/10.1016/j.apcatb.2022.121698

[S11] Y. Wu, H. Ding, T. Yang, Y. Xia, H. Zheng et al., Composite $NiCO_2O_4$@CeO_2 microsphere as cathode catalyst for high-performance lithium-oxygen battery. Adv. Sci. **9**(17), 2200523 (2022). https://doi.org/10.1002/advs.202200523

[S12] P. Wang, C. X. Li, S. H. Dong, X. L. Ge, P. Zhang et al., Hierarchical $NiCO_2S_4$@NiO core-shell heterostructures as catalytic cathode for long-life Li-O_2 batteries. Adv. Energy Mater. **11**(19), 2 (2021). https://doi.org/10.1002/aenm.202100799

[S13] S. Ma, Y. Lu, X. Zhu, Z. Li, Q. Liu, Efficient modulation of electron pathways by constructing a MnO_{2-x}@CeO_2 interface toward advanced lithium-oxygen batteries. ACS Appl. Mater. Interfaces **14**(19), 22104-22113 (2022). https://doi.org/10.1021/acsami.2c02318

[S14] P. Tan, W. Shyy, M. C. Wu, Y. Y. Huang, T. S. Zhao, Carbon electrode with nio and RuO_2 nanoparticles improves the cycling life of non-aqueous lithium-oxygen batteries. J. Power Sources **326**, 303-312 (2016). https://doi.org/10.1016/j.jpowsour.2016.07.012

[S15] X. Hu, F. Cheng, N. Zhang, X. Han, J. Chen, Nanocomposite of Fe_2O_3@c@MnO_2 as an efficient cathode catalyst for rechargeable lithium-oxygen batteries. Small. **11**(41), 5545-5550 (2015). https://doi.org/10.1002/smll.201501570

[S16] W. Wang, J. Cai, H. Wan, W. Cai, Z. Zhu et al., Electron-redistributed ni–co oxide nanoarrays as an orr/oer bifunctional catalyst for low overpotential and long lifespan Li–O_2 batteries. J. Mater. Chem. A **10**(27), 14613-14621 (2022). https://doi.org/10.1039/d2ta03604e

[S17] S.-S. Li, X.-L. Zhao, Y.-S. Liu, J.-J. Liu, K.-X. Wang et al., Tailoring the nucleation and growth routes of discharge products for lithium-oxygen batteries through the facet engineering of Ni2P catalysts. Energy Storage Mater. **56**, 506-514 (2023). https://doi.org/10.1016/j.ensm.2023.01.023

[S18] W. W. Wang, H. Wan, W. L. Cai, Y. T. Fang, Z. C. Fan et al., The free-standing n-coo matrix towards optimizing dual-electrodes for high-performance Li-O_2 batteries. Chem. Eng. J. **461**, 9 (2023). https://doi.org/10.1016/j.cej.2023.142004

[S19] K. Chen, G. Huang, J. L. Ma, J. Wang, D. Y. Yang et al., The stabilization effect of CO_2 in lithium–oxygen/CO_2 batteries. Angew. Chem. Int. Ed. **59**(38), 16661-16667 (2020). https://doi.org/10.1002/anie.202006303

[S20] L.-N. Song, L.-J. Zheng, X.-X. Wang, D.-C. Kong, Y.-F. Wang et al., Aprotic lithium–oxygen batteries based on nonsolid discharge products. J. Am. Chem.

Soc. **146**(2), 1305-1317 (2024). https://doi.org/10.1021/jacs.3c08656

[S21] G. Qi, J. Zhang, J. Cheng, B. Wang, Freestanding Mo_3N_2 nanotubes for long - term stabilized 2e− intermediate - based high energy efficiency Li–CO_2 batteries. SusMat. **3**(2), 276-288 (2023). https://doi.org/10.1002/sus2.123

[S22] L. Chen, J. Zhou, Y. Wang, Y. Xiong, J. Zhang et al., Flexible, stretchable, water-/fir-proof fiber-shaped Li-CO_2 batteries with high energy density. Adv Energy Mater. **13**(1), 2202933 (2022). https://doi.org/10.1002/aenm.202202933

[S23] L. J. Zheng, L. N. Song, X. X. Wang, S. Liang, H. F. Wang et al., Intrinsic stress-strain in barium titanate piezocatalysts enabling lithium−oxygen batteries with low overpotential and long life. Angew. Chem. Int. Ed. **62**(44), e202311739 (2023). https://doi.org/10.1002/anie.202311739

[S24] L. Ren, R. Zheng, D. Du, Y. Yan, M. He et al., Optimized orbital occupancy of transition metal in spinel ni-co oxides with heteroatom doping for aprotic Li-O_2 battery. Chem. Eng. J. **430**, 132977 (2022). https://doi.org/10.1016/j.cej.2021.132977

[S25] D. Li, J. Liang, S. J. Robertson, Y. Chen, N. Wang et al., Heterogeneous bimetallic organic coordination polymer-derived CO/Fe@NC bifunctional catalysts for rechargeable Li–O_2 batteries. ACS Appl. Mater. Interfaces **14**(4), 5459-5467 (2022). https://doi.org/10.1021/acsami.1c22643

[S26] Q. Lv, Z. Zhu, Y. Ni, B. Wen, Z. Jiang et al., Atomic ruthenium-riveted metal–organic framework with tunable d-band modulates oxygen redox for lithium–oxygen batteries. J. Am. Chem. Soc. **144**(50), 23239-23246 (2022). https://doi.org/10.1021/jacs.2c11676

[S27] W. L. Bai, Z. Zhang, K. X. Wang, J. S. Chen, Tuning discrete growth of ultrathin nonstoichiometric $Li_{2-x}O_2$ discs to achieve high cycling performance Li–O2 battery. Battery Energy **1**(4), 20220019 (2022). https://doi.org/10.1002/bte2.20220019
